# *Xylopia aethiopica* Seeds from Two Countries in West Africa Exhibit Differences in Their Proteomes, Mineral Content and Bioactive Phytochemical Composition

**DOI:** 10.3390/molecules24101979

**Published:** 2019-05-23

**Authors:** Xiaojian Yin, María A.S.C. Chávez León, Richard Osae, Loveth O. Linus, Lian-Wen Qi, Raphael N. Alolga

**Affiliations:** 1 State Key Laboratory of Natural Medicines, Department of Pharmacognosy, Institute of Pharmaceutical Science, China Pharmaceutical University, Nanjing 210009, China; ajian.517@163.com; 2 State Key Laboratory of Natural Medicines, Department of Pharmacognosy, School of Traditional Chinese Pharmacy, China Pharmaceutical University, No. 639 Longmian Avenue, Nanjing 211198, China; shengchuleon@gmail.com (M.A.S.C.C.L.); lovethlinus758@yahoo.com (L.O.L.); 3 School of Food and Biological Engineering, Jiangsu University, Zhenjiang 212013, China; osaerichard53@yahoo.com

**Keywords:** *Xylopia aethiopica*, Ghana, Nigeria, protein profile, mineral content, bioactive phytochemical composition

## Abstract

Aside from its multiple medicinal uses, the fruit of *Xylopia aethiopica* is widely used in Africa as food. Herein, we characterize the protein profiles, mineral content and bioactive phytochemical composition of the seeds of this plant sourced in Ghana and Nigeria. Using label-free proteomics, a total of 677 proteins were identified, with 260 found in the Ghana-sourced samples while 608 proteins were detected in the samples from Nigeria. However, 114 proteins were common between the samples from the two countries, among which 48 were significantly changed. Bioinformatics and functional analyses revealed that the differential levels of the proteins were mainly linked to pathways involved amino acids metabolism and biosynthesis. The significantly changed proteins related mainly to catalytic activity and carbon metabolism. The samples from Nigeria also exhibited superior qualities in terms of their antioxidant effects, and total phenolic and flavonoid content. Finally, only the content of Na varied to a statistically significant level. This study lends support to its culinary use and hints towards the impact of location of cultivation on the quality of the seeds. There is however need for further mechanistic investigations to unravel the underlying reasons for the observed differences.

## 1. Introduction 

*Xylopia aethiopica* (Dunal) A. Rich, family Annonaceae is a tall, slim, aromatic, evergreen tree that grows to 15–30 m high and 60–70 cm in diameter [1]. It is known to naturally grow in the Savanna region of Africa, particularly in Ghana, Nigeria, Cameroon, Ethiopia, and Senegal to name a few.

Known popularly as “Guinea pepper” or “Negro pepper,” the fruit of *X. aethiopica* has a wide range of documented uses in folkloric medicine in countries such as Ghana and Nigeria. It is used traditionally to treat female infertility, hemorrhoids, uterine fibroid, malaria, amenorrhea, cough, syphilis, diabetes, and dysentery among others. The seeds specifically are crushed and topically applied on the forehead to treat neuralgia and headache. The seeds when taken as a decoction or chewed treat epilepsy, numbness, and anemia. The seeds are also known to be used traditionally to enhance postpartum placental expulsion [1]. Following scientific scrutiny, a number of the purported traditional uses were proven. These include antiplasmodial [2,3] analgesic [4], anti-inflammatory [5], antidiabetic [6], and antimicrobial effects [7,8] among others. Aside from the aforementioned beneficial health benefits, the fruit of *X. aethiopica* is a well-known spice, used due to its rich nutritional value. 

It is known that the quality of plant organs such as fruits and seeds is influenced by a composite interaction between genetic and diverse environmental factors [9,10,11]. Plants respond to environmental influences such as abiotic stress in diverse ways. These interactions invariably impact on the genetic make-up of the plants with a consequential effect on the downstream products such as their cellular proteomes [12]. Also the area of cultivation impacts on the mineral content and phytochemistry of plant products including fruits and seeds [9]. Though a considerably good level of research has been done, to our knowledge there is no study on the proteome profile of *X. aethiopica* seeds. There is also no report on the probable influence of locations of cultivation and the inherent growth conditions on the mineral content and bioactive phytochemical composition of the fruit or seed of *X. aethiopica*.

Proteomics is defined as the high-throughput study of proteins. Proteomics via state-of-the-art quantitative techniques and bioinformatics enables identification, quantitation, validation, and characterization of a variety of proteins from a specific organ, tissue, or cell. The information obtained therein aside from aiding in determining protein structure, underlying enzymatic mechanisms, and regulatory functions, provides invaluable links between protein levels and stress tolerance [13,14]. It is also a reliable platform for analyzing gene response of non-model plants, especially those whose genome has not yet been fully sequenced. The major strength of proteomics in this regard is because it is centered on functional genomic translation.

Herein, we sought to comparatively characterize the proteome profile (for the first time), mineral content, and bioactive phytochemistry of the dried seeds of *X. aethiopica* from two countries in West Africa, Ghana, and Nigeria. Through this comparison, we sought to find scientific evidence to support its folkloric use as nutritious food in those countries. This manuscript, therefore, reports our findings obtained therefrom.

## 2. Results and Discussion

### 2.1. External Characteristics of X. Aethiopica Seeds and Fruits

**Figure 1:**
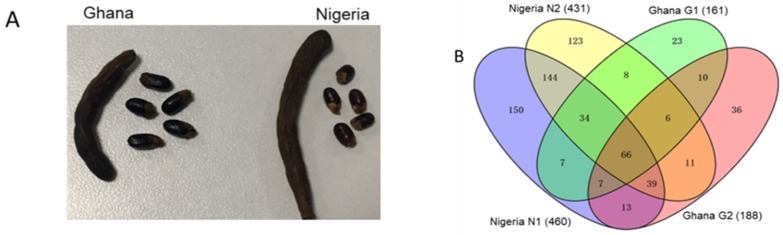
(**A**). Pictorial presentation of the phenotypic characteristics of *Xylopia aethiopica* fruits and seeds from Ghana and Nigeria. (**B**). Venn diagram presentation of protein distribution including common proteins identified in the seeds of *X. aethiopica* from Nigeria and Ghana. Proteins extracted from seeds were digested, analyzed using nano-LC-MS/MS, and identified based on protein ID number.

An examination of the seeds reveals that they are kidney-shaped, black in color, approximately 10 mm in length, and each pod usually contains between 5 to 8 seeds. The fruits are usually a dense cluster of small carpels (about 7–24, not shown), twisted bean-like pods, cylindrical, dark brown in color with visible external contours of the seeds as shown in Figure 1A. The external phenotypic characteristics of the fruits and seeds of *X. aethiopica* from the two countries were similar but quite distinguishable in terms of size and color. Those from Ghana were generally darker in color and slightly bigger. However, the pods of the Nigeria-sourced samples were bigger and contained more seeds.

### 2.2. Proteomics

#### 2.2.1. Total Number of Proteins and Significantly Changed Common Proteins

A comparative assessment of the protein profile of *X. aethiopica* seeds from Ghana and Nigeria via the label-free proteomics yielded a total of 677 proteins. Of these, 260 proteins were identified in the samples from Ghana while 608 were found in the seeds obtained from Nigeria (Supplementary Tables S1 and S2). A total of 114 proteins, however, were found to be common among the samples from the two countries (Figure 1B). Among the identified proteins, the levels of 48 were found to be significantly different with a *p*-value < 0.05 (Table 1). The number of proteins identified only in the Ghana-sourced samples was 69 while that for the Nigeria-sourced samples was 417. These differences could be partly ascribed to the different geographical locations and the inherent growth conditions. Also, the phenotypic differences could be a contributory factor. However, the exact cause(s) require further studies. A cursory look at the significantly changed proteins reveals that most of them are still uncharacterized including the top 4 with change ratios ranging from 44.50 to 24.00 (Table 1). Therefore the most abundant of the characterized proteins from this group include heat shock protein90-2 (HSP90-2), tubulin β-chain, malic enzyme, and clathrin heavy chain. The HSP90 and its isoforms play pivotal roles in the plant’s response to abiotic stress as well in plant immunity [15,16]. The microtubules which are heterodimeric polymers of αβ-tubulin, are reported to provide shape to cells, maintain tracks for vesicle transport and segregation of chromosomes. They also play various crucial roles in the plant’s tolerance to abiotic stress [17]. Malic enzyme is known to play diverse roles in the plant notably in its involvement in seed germination and development as well as in the plant’s response to various external stresses [18,19]. The clathrin heavy chain subunits were reported to influence stomatal function, plant growth, and immunity [20,21].

#### 2.2.2. Functional Categorization

**Figure 2:**
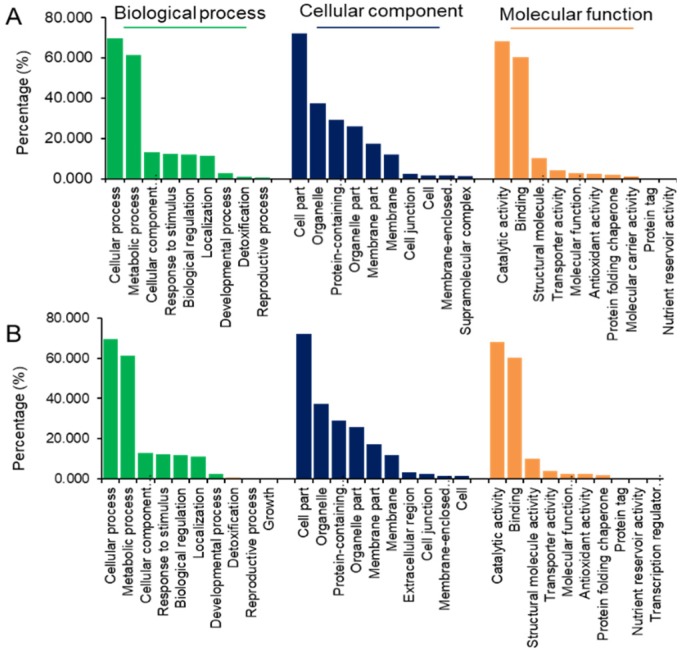
Functional categorization of identified proteins using the gene ontology (GO) database. The protein sequence was blasted against the GO database using blast2go v4.5 pipeline software. The protein function was predicted in categories including biological process, cell component, and molecular function. (**A**) Functional categorization of proteins identified from Nigeria-sourced samples. (**B**) Functional categorization of proteins identified from Ghana-sourced samples.

Since there is no information about genome sequence and gene annotation of *X. aethiopica*, functional analysis was performed by blasting the identified proteins against soybean based on peptides sequence similarity. Soybean is a known source of protein which grows in a range of climatic conditions. There have been several studies on it such as the influence of abiotic stress on its growth, development, and quality of seeds [22]. The soybean is also one to which much of its entire genome is sequenced, making it another model plant for research in plant biology beside *Arabidopsis thaliana*. After blasting, the molecular functions of the proteins were identified using gene ontology (GO) enrichment analysis. The proteins identified from all samples were broadly involved in biological process, cell component, and molecular function (Figure 2).

**Figure 3:**
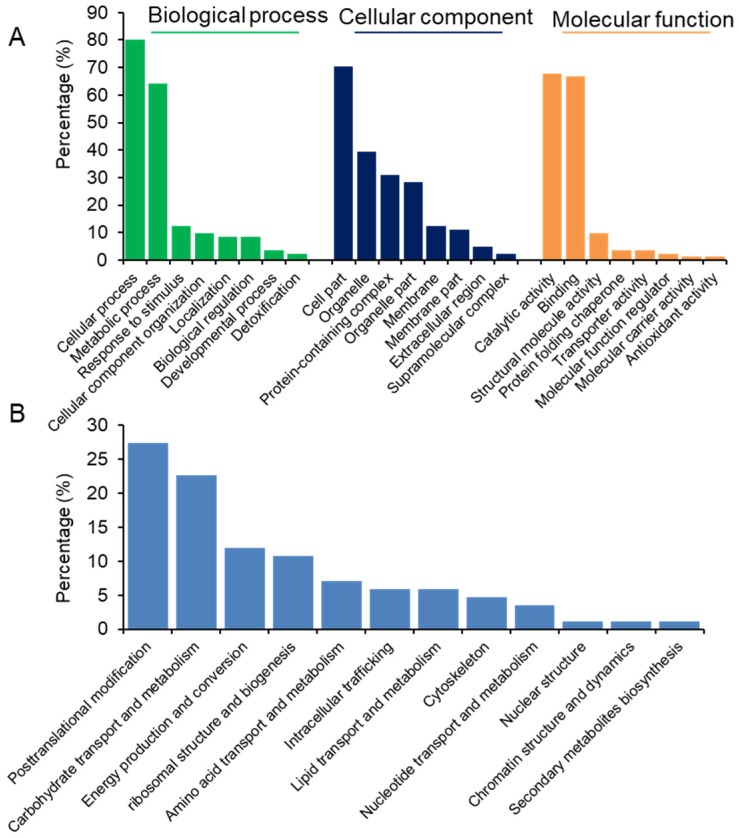
Functional analysis of significantly changed proteins between the seeds of *X. aethiopica* from Ghana and Nigeria. (**A**) Functional categorization of significantly changed proteins was analyzed using GO database with blast2go v4.5 pipeline software. (**B**) The molecular function of significantly changed proteins was analyzed using Clusters of Orthologous Groups of Proteins System (COG, http://www.ncbi.nlm.nih.gov/COG/).

With respect to the significantly changed proteins, aside from their broad functional categorization (biological process, cellular component, and molecular function), they are involved in a wide array of activities at the molecular function level. These activities range from posttranslational modification and carbohydrate transport to secondary metabolites biosynthesis (Figure 3).

#### 2.2.3. Pathway Enrichment Analysis

**Figure 4:**
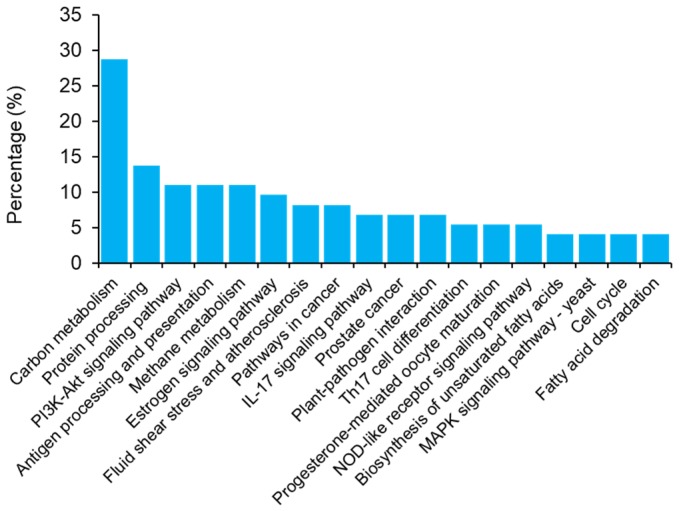
Pathway enrichment analysis of significantly changed proteins. The pathway analysis was performed using the KEGG database. The main related pathways accounting for the differential levels of the proteins are summarized.

KEGG pathway enrichment analysis of the significantly changed proteins revealed differences in 18 pathways ranging from carbon metabolism to fatty acid degradation (Figure 4). In summary, these pathways throw light on the various processes relating mainly to the biosynthesis and metabolism of amino acids. The differential expressions of these pathways by the samples from the two countries could be indicative of the possible influence of the location of cultivation and the growth conditions inherent therein. For instance, using the common pathway, carbon metabolism, the differential levels expressed by the samples suggests either varying levels of CO_2_ or significant differences in utilization of the CO_2_ during photosynthesis or the combination of both [23]. Either way, it points to the differential expressions and regulations of the genes involved in carbon metabolism and photosynthesis [24].

### 2.3. Elemental Analysis

With the aim of assessing the differences in the mineral content of *X. aethiopica* seeds from the two locations, 7 elements were quantified. As evidenced from Table 2, though there were differences in the average amounts of the 7 elements, these were not statistically significant except for Na (*p*-value < 0.05). These results hint towards the differential accumulation of these elements from the soil. This finding could also point to the differential levels of these elements in the cultivation sites of the two countries─further studies are however required to confirm this or otherwise.

### 2.4. Phytochemical Analysis

#### 2.4.1. Total Phenolic Content (TPC)

From Table 3, it is obvious that there is a significant difference (*p* < 0.05) in the TPC of the *X. aethiopica* samples gotten from the two countries. The TPC values for the samples of Ghanaian origin range from 49.61 to 67.34 mg GAE/g while that of the Nigeria-sourced samples is 79.65 to 84.85 mg GAE/g. The disparate TPC levels lend credence to the impact of external conditions such as climate, growing conditions, harvesting, and postharvest factors on the quality of plants (whether botanicals or not). This observation is largely congruent with other studies reported for other plant species. For instance, Zhang et al., (2018) reported the differential contents of alkaloids, polyphenols, and antioxidants in various cultivars of mulberry planted in different areas in Eastern China [25]. 

#### 2.4.2. Total Flavonoids Content (TFC)

The outcome of the TFC test (Table 3) found the Nigeria-sourced samples to possess higher levels of total flavonoids with values ranging from 90.03 to 95.59 mg CE/g while a range of 65.46 to 77.39 mg CE/g was realized for the samples from Ghana. This observation is congruent with that of Papoulias et al., (2009) who reported the influence of various factors such as genetic, preharvest, and postharvest factors on the content of bioactive compounds in white asparagus spears [26]. 

#### 2.4.3. Antioxidant Activities

The antioxidant effects of the various samples were assessed with four methods─ABTS, CUPRIC, DPPH, and FRAP methods. As evidenced from Table 3, a consistent trend is observed in the comparative antioxidant effects of the samples from two countries using all four methods. The samples from Nigeria exhibited superior antioxidant activities over those from Ghana regardless of the method used. The range of values for the Nigeria-sourced samples are as follows: ABTS, 39.53–46.40 mg/g db; CUPRIC, 60.35–80.20 mg/g db; DPPH, 99.53–107.7 mg/g db; FRAP, 83.50–89.70 mg/g db. The values obtained for the samples from Ghana are: ABTS, 18.15–36.70 mg/g db; CUPRIC, 26.72–36.96 mg/g db; DPPH, 82.39–89.20 mg/g db; FRAP, 55.57–66.84 mg/g db. These findings lend support to an earlier reported proposition─the higher the TPC value, the greater the antioxidant activity [27]. The differential antioxidant activities of samples from Ghana and Nigeria is also in tandem with reports by other researchers on the impact of geographical location or habitat on the chemical composition and content of bioactive compounds in plant samples [28,29].

## 3. Materials and Methods

### 3.1. Plant Material and Chemicals

Dried fruits of *X. aethiopica* were bought from the local producers in Tuobodom, Techiman, Ghana and Gombe, Nigeria in August 2017. The samples were identified by Dr. Raphael N. Alolga, voucher numbers assigned and deposited at the Department of Pharmacognosy, China Pharmaceutical University. Analar grade nitric acid was obtained from Nanjing Chemical Reagent Corp. (Nanjing, China). Water for analysis was purified using the Millipore Milli Q-Plus system (Millipore, Bedford, MA, USA). Standard stock solutions of the various elements were obtained from Merck, Darmstadt, Germany.

### 3.2. Proteomics

#### 3.2.1. Extraction of Protein

The extraction of proteins from the seeds of *X. aethiopica* prior to quantification was done according to the method earlier reported by Zhang et al. [30]. Briefly, a portion (0.1 g) of the seed was ground in liquid nitrogen and lysed in a solution containing 200 μL of L3 buffer (50 mM Tris-HCl, 7 M urea, 2 M thiourea, and 1× protease inhibitors cocktails), 800 μL of ice-cold acetone, and 10 mM dithiothreitol. The suspensions were incubated at −20 °C for 2 h. After centrifugation at 13,000× *g* for 20 min at 4 °C, the precipitated pellets were resuspended in 800 μL of ice-cold acetone containing 10 mM dithiothreitol. The suspensions were further centrifuged at 13,000× *g* for 20 min at 4 °C to collect the precipitated pellets and vacuum dried. The dried pellets were dissolved in 200 μL of L3 buffer. 

#### 3.2.2. Protein Quantification and Digestion

The concentration of the protein was determined using the Bradford method with bovine serum albumin as the standard [31]. The protein concentration was measured using the Bradford method with bovine serum albumin as the standard (Bradford, 1976). A portion (0.15 mg) of the protein sample was reduced with 10 mM dithiothreitol at 56 °C for 30 min, alkylated with 50 mM iodoacetamide for 30 min in the dark, and then diluted 4 times with 10 mM TEAB. Trypsin was added at an enzyme-protein ratio of 1:50 (*w/w*), and the reaction was performed at 37 °C for 16 h. After digestion, peptides were desalted using C18 columns, and the desalted peptides were dried with vacuum concentration meter. 

#### 3.2.3. Proteomic Data Processing

The raw MS/MS data were analyzed using Mascot Software v2.3. Proteins were identified based on a search against the soybean peptide database (54175 sequences) obtained from the soybean genome database (Phytozome version 9.1, http://www.phytozome.net/soybean) using the MASCOT search engine. The parameters used in the MASCOT search were the same as that employed by Yin et al. An automatic decoy database search was also performed. The results of the MASCOT search were subjected to analysis using the Percolator function in Protein Discover to enhance the accuracy and sensitivity of the identification process [32]. False discovery rates of <1% were used for the whole process of peptide identification. The MASCOT results generated by Protein Discover were used for differential analysis.

To establish the biological and functional properties of all proteins identified, their sequences were mapped with Gene Ontology (GO) Terms (http://geneontology.org/). The e-value was set to less than 1 × e^−5^, and the best hit for each query sequence was used for GO term matching. The GO term matching was performed with blast2go v4.5 pipeline. Clusters of Orthologous Groups of Proteins System (COG, http://www.ncbi.nlm.nih.gov/COG/) was employed for the molecular functional annotation of protein. Pathway mapping of identified proteins was performed using the KEGG database (http://www.genome.jp/kegg/). To determine the protein abundance in the samples, emPAI (exponentially modified protein abundance index) was applied [33]. Significantly changed proteins were those whose levels in the samples from the two countries were statistically significant (*p*-value < 0.05). The relative abundance of the proteins was determined by a ratio of the average peptide spectrum matching (PSM) of the samples from Nigeria to those from Ghana. 

### 3.3. Quantitative Elemental Analysis

#### 3.3.1. Sample Preparation (Microwave Acid-Assisted Digestion)

The seeds of each batch were milled, sieved, and a 0.1 g portion weighed into Teflon vessels. A 2 mL aliquot of 65% HNO_3_ was added and the mixture heated in an Advanced Microwave Labstation (Milestone, Microwave Laboratory systems) at a temperature of 160 °C for 1 h. Upon complete digestion, the clear solutions were diluted with distilled water to a final volume of 25 mL. For comparison, a blank solution of HNO_3_ was used. 

#### 3.3.2. Inductively Coupled Plasma-Optical Emission Spectrometric (ICP-OES) Analysis

The levels of Zn, P, Fe, Mg, Ca, Na, and K were assessed using an optical emission spectrometer (Optima 8000-PerkinElmer) with the following parameters: frequency, 27.12 MHz; RF Power, 1300 W; demountable quartz torch, Ar/Ar/Ar; coolant gas flows Ar, 12.0 L∙min^−1^; auxiliary gas Ar, 0.2 L∙min^−1^; nebulizer gas Ar, 0.55 L∙min^−1^; nebulizer pressure, 2.4 bar; glass spray chamber according to Scott, sample pump flow rate, 1.5 mL∙min^−1^; observation height 11 mm; holographic grating, 2400 grooves mm^−1^; dispersion of grating in the first reciprocal order, 0.55 nm mm^−1^; wavelength range of monochromator 165–800 nm. The wavelengths for Zn, P, Fe, Mg, Ca, Na, and K were respectively 206.200, 213.617, 238.204, 285.213, 317.933, 589.592, and 766.490 nm.

The 7 elements were grouped in three before analysis. The first group consisted of the most abundant elements. Standard solutions of Ca, Na, and K were prepared in different concentrations 10, 50, and 100 (mg/L) in 5 mL volumetric flasks. The second group included the trace elements, Zn, Fe, and Mg. Final concentrations of 0, 0.2, 0.5, 1, 2, and 5 (mg/L) were prepared in 10 mL volumetric flasks for these elements. Finally, P was prepared to final concentrations of 10 and 100 (mg/L) in 5 mL volumetric flasks and analyzed. All analyses were done in triplicate. The prepared standard solutions were used to draw calibration graphs from which the equations of the line and regression coefficients were obtained. The concentrations (in mg/g) of the various elements present in the various *X. aethiopica* samples were determined. 

### 3.4. Phytochemical Analysis

#### 3.4.1. Sample Preparation

To 1 g of each powdered sample weighed, a 20 mL aliquot of 80% methanol was added. The mixture was then ultrasonicated for 10 min (Trans-O-Sonic/D150-IM, Mumbai, India) and centrifuged (Hanil, Supra 22K, Korea) at 10,000× *g* for 30 min at 4 °C. The supernatants were then used for phytochemical determinations.

#### 3.4.2. Total Phenolic Content Determination (TPC)

TPC was determined with reference to the method of Jelled et al. (2015) with slight modification [34]. Folin–Ciocalteu reagent was diluted with distilled water at a ratio of 1:10 *v/v*. A 1 mL portion of the *X. aethiopica* solution was mixed with 5 ml Folin reagent. A 4 mL portion (75 g/L) of sodium carbonate was added, vortexed for 10 min and kept for 30 min at a temperature of 25 °C. Standard gallic acid solutions were used to develop the standard curve (R^2^ = 0.9979) and the absorbance of reaction mixture determined at 760 nm using a spectrophotometer, Model TU-1810 (Purkinje Instrument Ltd., Beijing, China). The methanol (80%) was used as the blank. The results obtained were expressed as mg of gallic acid equivalents (GAE) per gram of dry weight of each sample.

#### 3.4.3. Total Flavonoid Content Determination (TFC)

TFC was estimated according to the protocol described by Jelled et al. with slight changes [34]. Briefly, 0.5 mL of the supernatant, 2 mL of distilled water, and 0.15 mL of NaNO_2_ solution (5%) were mixed and kept for 6 min. AlCl_3_ solution (10%, 0.15 mL) was then added and the mixtures again left for 6 min before adding NaOH solution (4%, 2 mL). Distilled water was immediately added to the final desired volume of 5 mL, and the mixtures left for 15 min prior to determining their absorbances at 510 nm using a spectrophotometer, Model TU-1810 (Purkinje Instrument Ltd., Beijing, China). The results were calculated as mg of catechin equivalents (CE) per gram of dry weight of each sample. 

#### 3.4.4. Antioxidant Activities

Evaluation of the antioxidant activities of the extracts was performed in accordance with the methods of Li et al. with minor changes [35]. The DPPH (1,1-diphenyl-2-picrylhydrazyl) and ABTS (2,2-azino-bis-(3-ethylbenzothiazoline-6-sulfonic acid), CUPRIC (cupric ion reducing capacity) and FRAP (ferric reducing antioxidant power) methods were employed to assess the antioxidant activities of the *X. aethiopica* samples. The results of studies (i.e., ABTS, CUPRIC, DPPH, FRAP) were expressed as mg of Trolox equivalent per gram of sample on a dry basis.

##### ABTS

A 125 µL portion of the supernatant was mixed with 5 mL of ABTS solution (composed of 2.45 mM ABTS in ammonium persulfate incubated in darkness for 16 h). This was then kept for 15 min at 25 °C and the absorbance was taken at 734 nm.

##### Cupric Ion Reducing Capacity (CUPRIC)

To a 100 μL aliquot of the supernatant was added 4 mL of a solution composed of neocuproine (7.5 mM), copper(II) chloride (10 mM), ammonium acetate (1 M) and distilled water (1:1:1:1). The resultant mixture was then kept for 60 min at 25 °C prior to determining its absorbance at 450 nm.

##### DPPH Radical Scavenging Activity

A 6 mL volume of DPPH solution (60 mM in methanol) was mixed with 1 mL aliquot of the supernatant, left in a dark chamber for 30 min at 25 °C and the absorbance determined at 517 nm. 

##### Ferric Reducing Antioxidant Power Capacity (FRAP)

The supernatant (200 μL) was mixed with 6 mL of a solution made up of acetate buffer (300 mM, pH 3.6), iron(III) chloride (20 mM), TPTZ (10 mM in 40 mM HCl) (1:10:1), and distilled water (600 μL). This was then kept at 37 °C for 30 min and its absorbance was recorded at 593 nm.

### 3.5. Statistical Analysis

Data from the ICP-OES and phytochemical analyses are presented as the mean ± standard deviation values. A comparison of the differential levels of the 7 elements and various phytochemicals was performed by one-way analysis of variance (ANOVA) with the Dunnett’s post hoc test using SPSS (IBM, version 20). *p*-values < 0.05 denoted significant differences.

## 4. Conclusions

In conclusion, this study to our best knowledge is the first to successfully profile the proteins in the dried seeds of *X. aethiopica* as well as establish the difference that exists in the proteome, mineral content and phytochemistry of *X. aethiopica* seeds from Ghana and Nigeria. The findings hint on the possible effect of the location of cultivation and the inherent growth conditions on the quality of the seeds. This study also provides scientific support at least in part to its use as a good source of nutrition. Aside from providing a glimpse into the proteome of the dried seeds of the plant, our study opens the door to a wide array of studies to be further conducted. For instance, this study provides a platform for studies into the proteome changes of the seeds during various stages of germination. There is also a need for molecular mechanistic studies to bring to clarity the underlying reason(s) for the observed differences. To ascertain these findings or otherwise, it is recommended to use a larger sample size as well as include samples from other parts of these two countries in future studies. 

## Figures and Tables

**Table 1:** The significantly changed proteins between the seeds of *X. aethiopica* from Ghana and Nigeria.

Protein ID	Description	PSM (Nigeria)		PSM (Ghana)		Ratio (Nigeria/Ghana)	*p*-Value
		N1	N2	G1	G2		
I1MQ89	Uncharacterized protein	51	38	1	1	44.500	0.022
I1K4W1	Uncharacterized protein	25	32	1	1	28.500	0.016
I1MPI1	Uncharacterized protein	31	22	1	1	26.500	0.030
I1MT10	Uncharacterized protein	25	23	1	1	24.000	0.002
D6C500	HSP90-2	26	19	1	1	22.500	0.025
I1JT28	Tubulin beta chain	15	11	1	1	13.000	0.027
I1KZK0	Uncharacterized protein	17	14	1	2	10.333	0.013
I1K5Y5	Malic enzyme	8	10	1	1	9.000	0.015
I1LMS9	Clathrin heavy chain	8	10	1	1	9.000	0.015
I1JY29	Uncharacterized protein	9	7	1	1	8.000	0.020
I1JWK3	Uncharacterized protein	9	6	1	1	7.500	0.049
I1M3L8	Uncharacterized protein	16	14	3	1	7.500	0.012
I1JPX8	Fructose-bisphosphate aldolase	8	7	1	1	7.500	0.006
A0A0R0GRY6	Uncharacterized protein	11	10	2	1	7.000	0.006
A0A0R0GJT8	Uncharacterized protein	6	8	1	1	7.000	0.027
I1NDI4	Uncharacterized protein	8	6	1	1	7.000	0.027
A0A0R0FJR0	40S ribosomal protein SA	8	6	1	1	7.000	0.027
I1NAI7	Uncharacterized protein	39	29	9	1	6.800	0.045
I1KEN4	Uncharacterized protein	16	14	1	4	6.000	0.020
I1MJU7	Uncharacterized protein	5	6	1	1	5.500	0.012
I1KU21	Uncharacterized protein	10	12	3	1	5.500	0.024
I1M5G8	Malic-enzyme	6	5	1	1	5.500	0.012
I1L0G9	Tubulin beta chain	20	15	3	4	5.000	0.032
A0A0R4J4C3	Elongation factor 1-alpha	17	20	7	1	4.625	0.050
C6T1V2	Uncharacterized protein	5	4	1	1	4.500	0.020
I1MQS6	EF1Bgamma class glutathione S-transferase	4	5	1	1	4.500	0.020
I1KPH6	6-phosphogluconate dehydrogenase	5	4	1	1	4.500	0.020
A0A0R0F2E8	Uncharacterized protein	7	6	1	2	4.333	0.019
I1KH24	Phospholipase D	5	6	1	2	3.667	0.030
K7KYB1	Aconitate hydratase	6	5	2	1	3.667	0.030
I1N0W4	Xyloglucan endotransglucosylase/hydrolase	3	4	1	1	3.500	0.038
I1JDR2	Uncharacterized protein	4	3	1	1	3.500	0.038
A0A0R0FB78	Uncharacterized protein	4	3	1	1	3.500	0.038
I1MC13	Uncharacterized protein	4	3	1	1	3.500	0.038
I1KZJ0	Uncharacterized protein	3	4	1	1	3.500	0.038
I1K672	Uncharacterized protein	4	3	1	1	3.500	0.038
I1JCQ7	Succinate dehydrogenase [ubiquinone]	3	4	1	1	3.500	0.038
I1JXI9	Proteasome subunit beta	4	3	1	1	3.500	0.038
I1JJM3	Fructose-bisphosphate aldolase	7	7	1	3	3.500	0.038
I1L314	Heat shock protein 90-1	36	27	8	11	3.316	0.043
I1LNM2	NADH dehydrogenase subunit 9	4	4	1	2	2.667	0.038
A0A0R0HEQ3	Histone H2B	4	4	2	1	2.667	0.038
C6TGU2	Proteasome subunit alpha type	6	7	2	3	2.600	0.030
O48548	Aspartate aminotransferase	5	4	2	2	2.250	0.038
I1MC31	Uncharacterized protein	19	18	10	10	1.850	0.003
A0A0R0HEM1	Uncharacterized protein	1	1	12	14	0.077	0.007
I1L3K7	Uncharacterized protein	1	1	13	16	0.069	0.012

The protein ID was obtained through blasting against a soybean protein database. “PSM” means peptide spectrum matching and was used to estimate the protein abundance.

**Table 2:** Content of Zn, P, Fe, Mg, Ca, Na, and K (mg/g) in representative batches of *X. aethiopica* from Ghana and Nigeria.

Element	Ghana				Nigeria				*p*-Value
	G1	G2	G3	Average	N1	N2	N3	Average	
Zn	0.052 ± 0.001	0.049 ± 0.016	0.037 ± 0.07	0.046	0.045 ± 0.10	0.032 ± 0.12	0.040 ± 0.09	0.039	0.304
P	1.197 ± 0.03	1.190 ± 0.06	0.713 ± 0.01	1.034	1.286 ± 0.21	0.595 ± 0.18	0.920 ± 0.13	0.934	0.717
Fe	1.721 ± 0.20	1.248 ± 0.08	0.771 ± 0.11	1.246	0.785 ± 0.11	0.452 ± 0.23	0.847 ± 0.31	0.695	0.140
Mg	10.505 ± 0.05	11.142 ± 0.01	4.835 ± 0.1	8.827	13.025 ± 0.19	5.318 ± 0.24	8.039 ± 0.14	8.794	0.992
Ca	3.270 ± 0.16	3.201 ± 0.20	1.839 ± 0.09	2.77	3.023 ± 0.35	1.772 ± 0.24	2.740 ± 0.28	2.512	0.689
Na	0.024 ± 0.03	0.222 ± 0.01	0.251 ± 0.07	0.165	0.326 ± 0.76	0.479 ± 0.81	0.499 ± 0.98	0.435	0.040 *
K	8.149 ± 0.09	6.760 ± 0.06	4.049 ± 0.02	6.319	4.878 ± 0.78	2.168 ± 0.89	3.772 ± 0.68	3.606	0.132

“G1, G2, G3” mean representative samples from Ghana. “N1, N2, N3” mean representative samples from Nigeria. * Statistically significant difference.

**Table 3:** Results of total phenolic and flavonoid content and antioxidant activities of representative *X. aethiopica* samples from Ghana and Nigeria.

Samples	Total Phenolic and Flavonoid Content		Antioxidant Activities			
	TPC (mg GAE/g)	TFC (mg CE/g)	ABTS (mg/g db)	CUPRIC (mg/g db)	DPPH (mg/g db)	FRAP (mg/g db)
G1	58.94 ± 8.54 ^a^	67.73 ± 3.39 ^ab^	18.15 ± 5.06 ^e^	32.44 ± 16.70 ^c^	89.20 ± 0.40 ^c^	66.84 ± 4.46 ^c^
G2	67.34 ± 7.47 ^a^	77.39 ± 0.77 ^ab^	20.33 ± 3.95 ^e^	26.72 ± 3.36 ^c^	82.39 ± 0.33 ^d^	62.11 ± 3.77 ^cd^
G3	49.61 ± 0.49 ^a^	65.46 ± 2.62 ^b^	36.70 ± 6.81 ^d^	36.96 ± 10.37 ^c^	83.73 ± 0.48 ^d^	55.57 ±10.08 ^d^
N1	84.85 ± 5.32 ^ab^	90.03 ± 4.21 ^a^	46.40 ± 3.63 ^a^	60.35 ± 2.67 ^ab^	107.07 ± 0.81 ^a^	83.50 ± 4.09 ^b^
N2	81.92 ± 7.96 ^bc^	95.59 ± 2.89 ^a^	39.53 ± 1.37 ^c^	79.89 ± 5.93 ^a^	99.53 ± 1.06 ^b^	87.27 ± 0.42 ^ab^
N3	79.65 ± 5.08 ^c^	92.5 ± 3.9 ^a^	42.89 ± 4.04 ^b^	80.20 ± 6.27 ^a^	104.105 ± 0.82 ^a^	89.70 ± 3.09 ^a^

“G1, G2, G3”: samples from Ghana. “N1, N2, N3”: Samples from Nigeria. The superscript of the various letters on their respective values signify significant differences (*p* < 0.05).

## Supplementary Materials

Proteins identified in the seeds of *X. aethiopica* from Ghana, Table S2: Proteins identified in the seeds of *X. aethiopica* from Nigeria.
