# Supplementary material for: Xylopia aethiopica Seeds from Two Countries in West Africa Exhibit Differences in Their Proteomes, Mineral Content and Bioactive Phytochemical Composition
Source: Molecules. 2019 May 23;24(10):1979. doi: 10.3390/molecules24101979 (PMC6572195; doi:10.3390/molecules24101979)
Supplement: Supplementary file 1 [file molecules-24-01979-s001.pdf]

Supplementary Material

Xylopia aethiopica Seeds from Two Countries in West Africa Exhibit Differences in Their Proteomes, Mineral Content and Bioactive Phytochemical Composition

Xiaojian Yin [1,†], María A.S.C. Chávez León[2,†], Richard Osae [3], Loveth O. Linus [2], Lian-Wen Qi [2,*] and Raphael N. Alolga [2,*]

[1] State Key Laboratory of Natural Medicines, Department of Pharmacognosy, Institute of Pharmaceutical Science, China Pharmaceutical University, Nanjing 210009, China; ajian.517@163.com

[2] State Key Laboratory of Natural Medicines, Department of Pharmacognosy, School of Traditional Chinese Pharmacy, China Pharmaceutical University, No. 639 Longmian Avenue, Nanjing 211198, China; shengchuleon@gmail.com (M.A.S.C.C.L.); lovethlinus758@yahoo.com (L.O.L.)

[3] School of Food and Biological Engineering, Jiangsu University, Zhenjiang 212013, China; osaerichard53@yahoo.com

* Correspondence: Qilw@cpu.edu.cn (L.-W.Q.); alolgara@cpu.edu.cn (R.N.A.);
Tel.: +86-159-5108-8310 (L.-W.Q.); +86-152-6148-5837 (R.N.A.)

† These two authors are the co-first authors.

Supplementary Table S1. Proteins identified in the seeds of *X. aethiopica* from Ghana

Protein ID	Description	Peptides (G1)	Peptides (G2)	Unique peptides (G1)	Unique peptides (G2)	PSMs (G1)	PSMs (G2)
I1KU21	Uncharacterized protein OS=Glycine max OX=3847 GN=100804251 PE=4 SV=1	1	N/A	1	N/A	3	N/A
A0A0R0K0U9	Uncharacterized protein OS=Glycine max OX=3847 GN=GLYMA_05G022200 PE=3 SV=1	1	1	1	1	1	1
I1LL24	Uncharacterized protein OS=Glycine max OX=3847 GN=100806471 PE=3 SV=1	1	2	1	2	1	3
A0A0R0GRY6	Uncharacterized protein OS=Glycine max OX=3847 GN=GLYMA_14G176900 PE=3 SV=1	1	N/A	1	N/A	2	N/A
I1KPH6	6-phosphogluconate dehydrogenase, decarboxylating OS=Glycine max OX=3847	1	N/A	1	N/A	1	N/A
I1LZ93	Isocitrate dehydrogenase [NAD] subunit, mitochondrial OS=Glycine max OX=3847	1	2	1	2	1	3
A0A0R0I373	Uncharacterized protein OS=Glycine max OX=3847 GN=GLYMA_09G032900 PE=3 SV=1	1	1	1	1	14	13
I1K2I1	Tubulin alpha chain OS=Glycine max OX=3847 GN=100787058 PE=3 SV=1	1	1	1	1	2	3
I1N8I7	Uncharacterized protein OS=Glycine max OX=3847 GN=GLYMA_19G121900 PE=4 SV=1	1	3	1	3	7	9
I1K3S4	Adenosylhomocysteinase OS=Glycine max OX=3847 GN=GLYMA_05G152000 PE=3 SV=1	1	N/A	1	N/A	1	N/A
A0A0R0EXX1	Pyruvate kinase OS=Glycine max OX=3847 GN=100781158 PE=3 SV=1	1	N/A	1	N/A	1	N/A
I1KD37	Uncharacterized protein OS=Glycine max OX=3847 GN=100816315 PE=4 SV=1	1	N/A	1	N/A	3	N/A
I1NAI6	Peroxiredoxin OS=Glycine max OX=3847 GN=100783307 PE=3 SV=1	1	N/A	1	N/A	2	N/A
I1MJU7	Uncharacterized protein OS=Glycine max OX=3847 GN=100800698 PE=3 SV=1	1	N/A	1	N/A	1	N/A
I1JDR2	Uncharacterized protein OS=Glycine max OX=3847 GN=100786978 PE=4 SV=1	1	1	1	1	1	1
I1KZJ9	Uncharacterized protein OS=Glycine max OX=3847 GN=100810476 PE=4 SV=1	1	N/A	1	N/A	1	N/A
I1JXI9	Proteasome subunit beta OS=Glycine max OX=3847 GN=100778498 PE=3 SV=1	1	1	1	1	1	1
I1K5G1	T-complex protein 1 subunit delta OS=Glycine max OX=3847 GN=100786888 PE=3 SV=1	1	N/A	1	N/A	1	N/A
I1LKU1	Uncharacterized protein OS=Glycine max OX=3847 GN=100794780 PE=3 SV=1	1	N/A	1	N/A	1	N/A
I1LJE2	Coatomer subunit gamma OS=Glycine max OX=3847 GN=100791801 PE=3 SV=1	1	1	1	1	1	1
A0A0R0L9T9	40S ribosomal protein S4 OS=Glycine max OX=3847 GN=100801792 PE=3 SV=1	1	1	1	1	1	2
I1JCQ7	Succinate dehydrogenase [ubiquinone] flavoprotein subunit, mitochondrial	1	1	1	1	1	1
I1KEY6	26S proteasome non-ATPase regulatory subunit 2 homolog 1	1	1	1	1	1	1
A0A0R0IU60	Uncharacterized protein OS=Glycine max OX=3847 GN=100777674 PE=3 SV=1	1	2	1	2	1	3
O48548	Aspartate aminotransferase OS=Glycine max OX=3847 GN=AAT PE=2 SV=1	1	1	1	1	2	2
I1KZJ0	Uncharacterized protein OS=Glycine max OX=3847 GN=100807267 PE=3 SV=1	1	1	1	1	1	1
A0A0R0IUS1	Serine hydroxymethyltransferase OS=Glycine max OX=3847 GN=100305380 PE=3 SV=1	1	1	1	1	1	3
A0A0R0I5Z8	Lactoylglutathione lyase OS=Glycine max OX=3847 GN=GLYMA_09G004300 PE=3 SV=1	1	1	1	1	3	3
I1KYF8	40S ribosomal protein S8 OS=Glycine max OX=3847 GN=100803006 PE=3 SV=1	1	1	1	1	3	6
I1NH45	Peroxidase OS=Glycine max OX=3847 GN=100775837 PE=3 SV=1	1	1	1	1	4	6
I1LNM2	NADH dehydrogenase subunit 9 OS=Glycine max OX=3847 GN=nad9 PE=3 SV=1	1	2	1	2	1	2

I1KWQ3	Uncharacterized protein OS=Glycine max OX=3847 GN=GLYMA_08G256600 PE=4 SV=1	1	1	1	1	1	2
I1LUJ6	Uncharacterized protein OS=Glycine max OX=3847 GN=100793227 PE=4 SV=1	1	1	1	1	1	1
K7N4X3	Alpha-galactosidase OS=Glycine max OX=3847 GN=100797088 PE=3 SV=1	1	2	1	1	2	9
I1K1X5	Proteasome subunit alpha type OS=Glycine max OX=3847 GN=100820387 PE=3 SV=1	1	N/A	1	N/A	1	N/A
B0M1A9	Peroxisomal 3-ketoacyl-CoA thiolase OS=Glycine max OX=3847 GN=100170734 PE=2 SV=1	1	1	1	1	1	1
I1NES8	Uncharacterized protein OS=Glycine max OX=3847 GN=GLYMA_20G090200 PE=3 SV=1	1	1	1	1	9	6
I1LAT2	Uncharacterized protein OS=Glycine max OX=3847 GN=100803952 PE=4 SV=1	1	1	1	1	3	2
I1MSH9	Signal peptidase I OS=Glycine max OX=3847 GN=100797179 PE=3 SV=1	1	1	1	1	1	1
I1K609	Uncharacterized protein OS=Glycine max OX=3847 GN=100787769 PE=3 SV=1	1	1	1	1	1	1
K7MAP3	Uncharacterized protein OS=Glycine max OX=3847 GN=GLYMA_15G102600 PE=3 SV=1	1	N/A	1	N/A	1	N/A
A0A0R0FQN0	Aspartate aminotransferase OS=Glycine max OX=3847 GN=547792 PE=4 SV=1	1	3	1	3	1	3
I1JBJ9	Endoglucanase OS=Glycine max OX=3847 GN=GLYMA_02G016400 PE=3 SV=1	1	N/A	1	N/A	1	N/A
I1MS58	Beta-galactosidase OS=Glycine max OX=3847 GN=GLYMA_17G047400 PE=3 SV=2	1	2	1	1	1	4
C6TGU0	DHAR class glutathione S-transferase OS=Glycine max OX=3847 GN=DHAR3 PE=2 SV=1	1	1	1	1	1	1
B0M1B1	Peroxisomal glycolate oxidase OS=Glycine max OX=3847 GN=100777153 PE=2 SV=1	1	N/A	1	N/A	1	N/A
I1J4G4	Profilin OS=Glycine max OX=3847 GN=100778045 PE=3 SV=1	1	1	1	1	2	1
I1M8A5	Proteasome subunit alpha type OS=Glycine max OX=3847 GN=100792921 PE=3 SV=1	1	N/A	1	N/A	1	N/A
A0A0R0J965	Uncharacterized protein OS=Glycine max OX=3847 GN=100809384 PE=4 SV=1	1	1	1	1	1	3
I1KL36	Uncharacterized protein OS=Glycine max OX=3847 GN=GLYMA_07G180100 PE=3 SV=1	1	N/A	1	N/A	1	N/A
I1LV40	Uncharacterized protein OS=Glycine max OX=3847 GN=100799923 PE=4 SV=1	1	1	1	1	1	1
I1L171	Uncharacterized protein OS=Glycine max OX=3847 GN=100800020 PE=4 SV=1	1	N/A	1	N/A	1	N/A
I1LFG4	Uncharacterized protein OS=Glycine max OX=3847 GN=100781676 PE=4 SV=1	1	N/A	1	N/A	1	N/A
A0A0R0F5Q2	Uncharacterized protein OS=Glycine max OX=3847 GN=100809809 PE=4 SV=1	1	N/A	1	N/A	1	N/A
C6T078	Uncharacterized protein OS=Glycine max OX=3847 GN=100306624 PE=2 SV=1	1	N/A	1	N/A	1	N/A
A0A0R4J3N1	Uncharacterized protein OS=Glycine max OX=3847 GN=100779787 PE=3 SV=1	1	N/A	1	N/A	1	N/A
C6TD38	Uncharacterized protein OS=Glycine max OX=3847 GN=100805113 PE=2 SV=1	1	1	1	1	1	3
K7KBZ4	Cysteine synthase OS=Glycine max OX=3847 GN=GLYMA_03G006700 PE=3 SV=1	1	N/A	1	N/A	1	N/A
I1K6N2	Uncharacterized protein OS=Glycine max OX=3847 GN=100805621 PE=4 SV=1	1	1	1	1	2	1
A0A0R0IY70	Serine/threonine-protein phosphatase 2A 55 kDa regulatory subunit B	1	N/A	1	N/A	1	N/A
C6SWA6	Histone H2A OS=Glycine max OX=3847 GN=100305705 PE=2 SV=1	1	1	1	1	2	1
I1JT85	Uncharacterized protein OS=Glycine max OX=3847 GN=100800985 PE=3 SV=1	1	N/A	1	N/A	1	N/A
I1KW20	Uncharacterized protein OS=Glycine max OX=3847 GN=100791896 PE=4 SV=1	1	1	1	1	1	1
K7MXZ0	GrpE protein homolog OS=Glycine max OX=3847 GN=100820521 PE=3 SV=1	1	N/A	1	N/A	1	N/A
I1NFX7	D-3-phosphoglycerate dehydrogenase OS=Glycine max OX=3847 GN=100808771 PE=3 SV=1	1	N/A	1	N/A	1	N/A
I1KH71	Acetyltransferase component of pyruvate dehydrogenase complex	1	N/A	1	N/A	1	N/A
A0A0R0HSV1	Uncharacterized protein OS=Glycine max OX=3847 GN=100800747 PE=3 SV=1	1	1	1	1	2	3

K7L1B9	Uncharacterized protein OS=Glycine max OX=3847 GN=GLYMA_07G108800 PE=4 SV=1	1	N/A	1	N/A	1	N/A
I1MSU1	Xylose isomerase OS=Glycine max OX=3847 GN=100804624 PE=3 SV=1	1	1	1	1	1	1
I1LZJ8	Uncharacterized protein OS=Glycine max OX=3847 GN=100791477 PE=3 SV=1	1	2	1	2	1	2
A0A0R0E8K1	Uncharacterized protein OS=Glycine max OX=3847 GN=100796915 PE=4 SV=1	1	1	1	1	1	1
I1JLY5	Uncharacterized protein OS=Glycine max OX=3847 GN=GLYMA_04G107900 PE=4 SV=1	1	1	1	1	1	1
I1LEK6	Uncharacterized protein OS=Glycine max OX=3847 GN=100781483 PE=4 SV=1	1	N/A	1	N/A	1	N/A
A0A0R0HJE4	Uncharacterized protein OS=Glycine max OX=3847 GN=GLYMA_11G194200 PE=4 SV=1	1	1	1	1	1	1
I1LJ68	Fructose-bisphosphate aldolase OS=Glycine max OX=3847 GN=100818915 PE=3 SV=1	1	N/A	1	N/A	1	N/A
I1LXQ1	Uncharacterized protein OS=Glycine max OX=3847 GN=100802266 PE=4 SV=2	1	N/A	1	N/A	1	N/A
C6SWX1	Uncharacterized protein OS=Glycine max OX=3847 GN=100499810 PE=2 SV=1	1	1	1	1	1	3
I1JMQ0	Nucleoside diphosphate kinase OS=Glycine max OX=3847 GN=GLYMA_03G109500 PE=3 SV=1	1	1	1	1	2	1
A0A0R0EVM7	Glutamine synthetase OS=Glycine max OX=3847 GN=GLYMA_18G041100 PE=3 SV=1	1	1	1	1	1	2
A0A0R0HR30	Uncharacterized protein OS=Glycine max OX=3847 GN=100500084 PE=4 SV=1	1	N/A	1	N/A	1	N/A
I1KG34	Uncharacterized protein OS=Glycine max OX=3847 GN=GLYMA_07G002300 PE=3 SV=1	1	2	1	2	1	2
I1L068	Uncharacterized protein OS=Glycine max OX=3847 GN=100784816 PE=4 SV=1	1	1	1	1	1	1
K7LWI4	ATP synthase subunit alpha OS=Glycine max OX=3847 GN=GLYMA_12G232000 PE=3 SV=1	1	2	1	1	2	3
I1KEA1	Uncharacterized protein OS=Glycine max OX=3847 GN=100779962 PE=4 SV=1	1	N/A	1	N/A	2	N/A
A0A0R0HLG7	Uncharacterized protein (Fragment) OS=Glycine max OX=3847	1	1	1	1	1	1
I1KGY6	Uncharacterized protein OS=Glycine max OX=3847 GN=100803691 PE=3 SV=1	1	N/A	1	N/A	1	N/A
I1L8L2	Uncharacterized protein OS=Glycine max OX=3847 GN=GLYMA_10G044000 PE=3 SV=1	1	1	1	1	2	1
A0A0R4J321	Proteasome subunit beta OS=Glycine max OX=3847 GN=100793551 PE=3 SV=1	1	N/A	1	N/A	1	N/A
I1KMS8	Uncharacterized protein OS=Glycine max OX=3847 GN=GLYMA_07G241600 PE=4 SV=1	1	N/A	1	N/A	1	N/A
K7K6W3	Uncharacterized protein OS=Glycine max OX=3847 GN=102662858 PE=4 SV=1	1	N/A	1	N/A	1	N/A
I1KXZ0	Phi class glutathione S-transferase OS=Glycine max OX=3847 GN=GSTF6 PE=2 SV=1	1	N/A	1	N/A	3	N/A
A0A0R0KN82	Uncharacterized protein OS=Glycine max OX=3847 GN=GLYMA_03G238900 PE=4 SV=1	1	1	1	1	5	12
I1LM73	UTP--glucose-1-phosphate uridylyltransferase OS=Glycine max OX=3847	1	1	1	1	2	2
K7LT57	Uncharacterized protein OS=Glycine max OX=3847 GN=100812560 PE=3 SV=1	1	N/A	1	N/A	1	N/A
I1LE01	Uncharacterized protein OS=Glycine max OX=3847 GN=100811411 PE=4 SV=1	1	N/A	1	N/A	2	N/A
I1JH86	Fructose-bisphosphate aldolase OS=Glycine max OX=3847 GN=GLYMA_02G222400 PE=3 SV=1	1	N/A	1	N/A	1	N/A
I1KFV5	Uncharacterized protein OS=Glycine max OX=3847 GN=GLYMA_06G321600 PE=3 SV=1	1	1	1	1	2	3
I1K4M0	Uncharacterized protein OS=Glycine max OX=3847 GN=100801361 PE=4 SV=1	1	1	1	1	1	1
I1MBZ5	Uncharacterized protein OS=Glycine max OX=3847 GN=100812416 PE=4 SV=1	1	N/A	1	N/A	1	N/A
I1MX58	Uncharacterized protein OS=Glycine max OX=3847 GN=100811577 PE=3 SV=1	1	N/A	1	N/A	1	N/A
A0A0R0GXN6	Uncharacterized protein OS=Glycine max OX=3847 GN=100793257 PE=3 SV=1	1	N/A	1	N/A	1	N/A
A0A368UH87	Uncharacterized protein OS=Glycine max OX=3847 GN=GLYMA_13G186000 PE=3 SV=1	1	N/A	1	N/A	1	N/A

I1MW49	Uncharacterized protein OS=Glycine max OX=3847 GN=100807833 PE=3 SV=2	1	1	1	1	1	1
D6QX28	Annexin 11 OS=Glycine max OX=3847 GN=ANN11 PE=2 SV=1	1	N/A	1	N/A	1	N/A
I1KC06	Uncharacterized protein OS=Glycine max OX=3847 GN=100813123 PE=4 SV=1	1	N/A	1	N/A	1	N/A
B0M1A1	Isocitrate lyase OS=Glycine max OX=3847 GN=ICL1 PE=2 SV=1	1	N/A	1	N/A	1	N/A
C6SV91	Proteasome subunit beta OS=Glycine max OX=3847 GN=100499664 PE=2 SV=1	1	N/A	1	N/A	1	N/A
I1JJF1	Uncharacterized protein OS=Glycine max OX=3847 GN=100808722 PE=3 SV=1	1	N/A	1	N/A	1	N/A
K7KBA9	Uncharacterized protein OS=Glycine max OX=3847 GN=GLYMA_02G283800 PE=3 SV=1	1	1	1	1	3	1
I1LPJ8	Uncharacterized protein OS=Glycine max OX=3847 GN=GLYMA_12G027300 PE=4 SV=1	1	N/A	1	N/A	1	N/A
I1LH25	Malate dehydrogenase OS=Glycine max OX=3847 GN=GLYMA_11G043900 PE=3 SV=1	1	N/A	1	N/A	1	N/A
I1KB92	Alpha-galactosidase OS=Glycine max OX=3847 GN=100784072 PE=3 SV=1	1	N/A	1	N/A	2	N/A
A0A0R0GX14	Uncharacterized protein OS=Glycine max OX=3847 GN=GLYMA_13G077700 PE=4 SV=2	1	N/A	1	N/A	1	N/A
K7K1V3	Uncharacterized protein OS=Glycine max OX=3847 GN=100783073 PE=4 SV=1	1	N/A	1	N/A	1	N/A
A0A0R0K565	Uncharacterized protein OS=Glycine max OX=3847 GN=100816263 PE=4 SV=1	1	N/A	1	N/A	1	N/A
I1L602	Uncharacterized protein OS=Glycine max OX=3847 GN=GLYMA_09G240600 PE=4 SV=1	1	N/A	1	N/A	1	N/A
I1M3L8	Uncharacterized protein OS=Glycine max OX=3847 GN=GLYMA_13G290700 PE=3 SV=2	2	N/A	2	N/A	3	N/A
I1L0G9	Tubulin beta chain OS=Glycine max OX=3847 GN=100818878 PE=3 SV=1	2	3	2	3	3	4
I1KTX8	Phosphoglycerate kinase OS=Glycine max OX=3847 GN=100795933 PE=3 SV=1	2	2	1	2	2	4
C6TGU2	Proteasome subunit alpha type OS=Glycine max OX=3847 GN=100796147 PE=2 SV=1	2	2	2	2	2	3
Q2I0H4	Glyceraldehyde-3-phosphate dehydrogenase OS=Glycine max OX=3847 GN=GAPC1 PE=2 SV=1	2	2	2	1	7	15
A0A0R0HEQ3	Histone H2B OS=Glycine max OX=3847 GN=100778668 PE=3 SV=1	2	1	2	1	2	1
K7MJY8	Sucrose synthase OS=Glycine max OX=3847 GN=GLYMA_17G045800 PE=3 SV=1	2	N/A	2	N/A	3	N/A
A0A0R0KN50	Uncharacterized protein OS=Glycine max OX=3847 GN=GLYMA_03G222100 PE=3 SV=1	2	2	2	2	3	2
A0A0R0HYT3	Uncharacterized protein OS=Glycine max OX=3847 GN=GLYMA_10G069600 PE=4 SV=1	2	N/A	2	N/A	3	N/A
A0A0R0LFB4	Dihydrolipoamide acetyltransferase component of pyruvate dehydrogenase complex	2	1	2	1	3	1
K7KYB1	Aconitate hydratase OS=Glycine max OX=3847 GN=100793264 PE=3 SV=1	2	1	1	1	2	1
I1JFW6	Uncharacterized protein OS=Glycine max OX=3847 GN=100803239 PE=3 SV=1	2	1	2	1	2	3
A0A0R4J4A9	Uncharacterized protein OS=Glycine max OX=3847 GN=100795325 PE=4 SV=1	2	3	2	2	4	5
I1JXP9	Uncharacterized protein OS=Glycine max OX=3847 GN=100796195 PE=3 SV=1	2	1	2	1	2	1
I1LRQ4	Aconitate hydratase OS=Glycine max OX=3847 GN=100778506 PE=3 SV=1	2	N/A	1	N/A	2	N/A
A0A0R0IRH4	Phosphoglycerate kinase OS=Glycine max OX=3847 GN=GLYMA_08G165400 PE=3 SV=1	2	N/A	1	N/A	2	N/A
I1J637	Uncharacterized protein OS=Glycine max OX=3847 GN=100788017 PE=4 SV=1	2	N/A	1	N/A	4	N/A
I1N1N3	Uncharacterized protein OS=Glycine max OX=3847 GN=100809646 PE=3 SV=2	2	1	2	1	2	1
I1KSD0	Tubulin alpha chain OS=Glycine max OX=3847 GN=GLYMA_08G115100 PE=3 SV=1	2	N/A	2	N/A	2	N/A
A0A0R0IXV4	Glucose-6-phosphate 1-dehydrogenase OS=Glycine max OX=3847 GN=100801945 PE=3 SV=1	2	N/A	2	N/A	2	N/A
A0A0R4J4C8	ATP synthase subunit beta OS=Glycine max OX=3847 GN=100797948 PE=3 SV=1	3	6	3	6	6	15

Q01915	ATP synthase subunit alpha, mitochondrial OS=Glycine max OX=3847 GN=ATPA PE=3 SV=1	3	5	3	4	8	7
A0A0R0FY49	Uncharacterized protein OS=Glycine max OX=3847 GN=GLYMA_15G088000 PE=3 SV=1	3	5	1	3	7	8
A0A0R4J4C3	Elongation factor 1-alpha OS=Glycine max OX=3847 GN=100776330 PE=3 SV=1	3	N/A	3	N/A	7	N/A
I1NAI7	Uncharacterized protein OS=Glycine max OX=3847 GN=GLYMA_19G190900 PE=3 SV=1	3	N/A	1	N/A	9	N/A
A0A0R4J2L9	Malate dehydrogenase OS=Glycine max OX=3847 GN=GLYMA_02G005500 PE=3 SV=1	3	3	3	3	3	6
I1L655	Uncharacterized protein OS=Glycine max OX=3847 GN=100814088 PE=3 SV=1	3	2	3	2	6	2
C6SZI8	Uncharacterized protein OS=Glycine max OX=3847 GN=100500031 PE=2 SV=1	3	N/A	3	N/A	4	N/A
A0A0R0F2Z4	Uncharacterized protein OS=Glycine max OX=3847 GN=100820155 PE=4 SV=1	3	1	3	1	3	1
I1MDV8	Uncharacterized protein OS=Glycine max OX=3847 GN=100817945 PE=3 SV=1	3	4	3	4	5	6
A0A0R0FDT6	Uncharacterized protein OS=Glycine max OX=3847 GN=100784882 PE=4 SV=1	3	N/A	2	N/A	4	N/A
A0A0R0KXC5	Uncharacterized protein OS=Glycine max OX=3847 GN=GLYMA_02G009600 PE=3 SV=1	3	3	3	3	4	5
A0A0R4J2M8	Proteasome subunit alpha type OS=Glycine max OX=3847 GN=100808731 PE=3 SV=1	3	2	3	2	4	4
I1KPN5	Uncharacterized protein OS=Glycine max OX=3847 GN=GLYMA_08G025900 PE=3 SV=2	3	5	1	3	6	13
I1KUR0	Uncharacterized protein OS=Glycine max OX=3847 GN=100817948 PE=3 SV=1	3	N/A	3	N/A	3	N/A
I1L314	Heat shock protein 90-1 OS=Glycine max OX=3847 GN=100811234 PE=2 SV=1	4	6	3	5	8	11
I1MC31	Uncharacterized protein OS=Glycine max OX=3847 GN=100779976 PE=3 SV=1	4	5	3	4	10	10
O23960	Biotin carboxylase OS=Glycine max OX=3847 GN=accC-2 PE=2 SV=1	4	3	4	3	5	5
A0A0R0EX12	Histone H4 OS=Glycine max OX=3847 GN=100779904 PE=3 SV=1	4	7	4	7	10	15
C6T4R9	Uncharacterized protein OS=Glycine max OX=3847 GN=100527579 PE=2 SV=1	4	5	4	5	7	16
I1J6K4	Uncharacterized protein OS=Glycine max OX=3847 GN=100808526 PE=3 SV=2	4	3	4	3	5	10
I1L3K7	Uncharacterized protein OS=Glycine max OX=3847 GN=100803925 PE=3 SV=1	4	5	2	3	13	16
A0A0R0HEM1	Uncharacterized protein OS=Glycine max OX=3847 GN=100813437 PE=3 SV=1	4	7	4	7	12	14
A0A0R4J4D6	Uncharacterized protein OS=Glycine max OX=3847 GN=100812707 PE=3 SV=1	6	N/A	2	N/A	12	N/A
A0A0R4J626	Uncharacterized protein OS=Glycine max OX=3847 GN=100777767 PE=3 SV=1	6	8	2	1	11	16
A0A0R0FH00	Uncharacterized protein OS=Glycine max OX=3847 GN=100787543 PE=3 SV=1	N/A	8	N/A	1	N/A	15
I1KZK0	Uncharacterized protein OS=Glycine max OX=3847 GN=GLYMA_08G363800 PE=3 SV=1	N/A	2	N/A	1	N/A	2
I1KEN4	Uncharacterized protein OS=Glycine max OX=3847 GN=100807407 PE=3 SV=1	N/A	4	N/A	2	N/A	4
K7L2A0	Uncharacterized protein OS=Glycine max OX=3847 GN=102667572 PE=3 SV=1	N/A	4	N/A	2	N/A	6
A0A0R0F2E8	Uncharacterized protein OS=Glycine max OX=3847 GN=GLYMA_19G228800 PE=3 SV=1	N/A	1	N/A	1	N/A	2
I1KH24	Phospholipase D OS=Glycine max OX=3847 GN=100794849 PE=3 SV=1	N/A	2	N/A	2	N/A	2
I1JY29	Uncharacterized protein OS=Glycine max OX=3847 GN=100783318 PE=3 SV=1	N/A	1	N/A	1	N/A	1
A0A0R0EHR6	6-phosphogluconate dehydrogenase, decarboxylating OS=Glycine max OX=3847	N/A	2	N/A	1	N/A	3
K7MJ32	Alpha-galactosidase OS=Glycine max OX=3847 GN=GLYMA_16G220400 PE=3 SV=1	N/A	3	N/A	2	N/A	9
C6SZX7	Glutathione peroxidase OS=Glycine max OX=3847 GN=100306570 PE=2 SV=1	N/A	1	N/A	1	N/A	1
I1JZP0	Uncharacterized protein OS=Glycine max OX=3847 GN=100783173 PE=4 SV=1	N/A	1	N/A	1	N/A	3
I1LUH6	Uncharacterized protein OS=Glycine max OX=3847 GN=100785990 PE=3 SV=1	N/A	2	N/A	2	N/A	2

I1JJM3	Fructose-bisphosphate aldolase OS=Glycine max OX=3847 GN=GLYMA_02G303000 PE=3 SV=1	N/A	2	N/A	2	N/A	3
I1JRK8	Proteasome subunit beta OS=Glycine max OX=3847 GN=GLYMA_03G244700 PE=3 SV=1	N/A	1	N/A	1	N/A	1
C6TD56	Glyceraldehyde-3-phosphate dehydrogenase OS=Glycine max OX=3847	N/A	2	N/A	1	N/A	6
A0A0R0JNS2	Uncharacterized protein OS=Glycine max OX=3847 GN=GLYMA_06G317400 PE=3 SV=1	N/A	1	N/A	1	N/A	2
I1K672	Uncharacterized protein OS=Glycine max OX=3847 GN=100805445 PE=4 SV=1	N/A	1	N/A	1	N/A	1
A0A0R4J3L5	Proteasome subunit alpha type OS=Glycine max OX=3847 GN=732640 PE=3 SV=1	N/A	1	N/A	1	N/A	1
A0A0R0FB78	Uncharacterized protein OS=Glycine max OX=3847 GN=100779679 PE=4 SV=1	N/A	1	N/A	1	N/A	1
Q42807	Stearoyl-[acyl-carrier-protein] 9-desaturase, chloroplastic OS=Glycine max OX=3847	N/A	1	N/A	1	N/A	1
I1JXC1	Elongation factor Tu OS=Glycine max OX=3847 GN=100805960 PE=3 SV=1	N/A	1	N/A	1	N/A	1
I1MSP4	Plasma membrane ATPase OS=Glycine max OX=3847 GN=100816905 PE=3 SV=2	N/A	1	N/A	1	N/A	1
A0A0R4J4X6	Uncharacterized protein OS=Glycine max OX=3847 GN=GLYMA_13G176300 PE=3 SV=1	N/A	2	N/A	2	N/A	2
C6T827	Pyruvate dehydrogenase E1 component subunit beta OS=Glycine max OX=3847	N/A	1	N/A	1	N/A	2
I1KHP5	Pyruvate dehydrogenase E1 component subunit alpha OS=Glycine max OX=3847	N/A	1	N/A	1	N/A	1
A5JVZ7	Superoxide dismutase OS=Glycine max OX=3847 GN=100785490 PE=2 SV=1	N/A	2	N/A	2	N/A	3
I1K467	Uncharacterized protein OS=Glycine max OX=3847 GN=100805255 PE=3 SV=1	N/A	1	N/A	1	N/A	1
C6TLW3	Uncharacterized protein OS=Glycine max OX=3847 GN=100809582 PE=2 SV=1	N/A	1	N/A	1	N/A	1
C6T4H4	Ribulose-phosphate 3-epimerase OS=Glycine max OX=3847 GN=100527490 PE=2 SV=1	N/A	1	N/A	1	N/A	1
C6TFG0	Uncharacterized protein OS=Glycine max OX=3847 GN=100810569 PE=2 SV=1	N/A	1	N/A	1	N/A	1
C6T4Z6	Uncharacterized protein OS=Glycine max OX=3847 GN=100527647 PE=2 SV=1	N/A	1	N/A	1	N/A	1
K7M4G7	Uncharacterized protein OS=Glycine max OX=3847 GN=GLYMA_14G020500 PE=4 SV=1	N/A	1	N/A	1	N/A	1
I1JZW5	Uncharacterized protein OS=Glycine max OX=3847 GN=GLYMA_05G033500 PE=3 SV=1	N/A	1	N/A	1	N/A	2
A0A0R0I7V5	Uncharacterized protein OS=Glycine max OX=3847 GN=GLYMA_10G224200 PE=3 SV=1	N/A	1	N/A	1	N/A	1
C6TGN8	Uncharacterized protein OS=Glycine max OX=3847 GN=100797023 PE=2 SV=1	N/A	1	N/A	1	N/A	1
A0A0R0FM59	Glucose-6-phosphate 1-dehydrogenase OS=Glycine max OX=3847 GN=100793462 PE=3 SV=1	N/A	1	N/A	1	N/A	1
O80412	Mitochondrial phosphate transporter OS=Glycine max OX=3847 GN=548006 PE=2 SV=1	N/A	1	N/A	1	N/A	1
I1N6R2	60S ribosomal protein L36 OS=Glycine max OX=3847 GN=100526979 PE=3 SV=1	N/A	1	N/A	1	N/A	1
A0A0R0EWX9	Uncharacterized protein OS=Glycine max OX=3847 GN=100791967 PE=4 SV=1	N/A	2	N/A	2	N/A	3
A0A0R0HHB6	Uncharacterized protein OS=Glycine max OX=3847 GN=GLYMA_11G146300 PE=3 SV=1	N/A	1	N/A	1	N/A	1
I1JGN1	Uncharacterized protein OS=Glycine max OX=3847 GN=100780041 PE=3 SV=1	N/A	1	N/A	1	N/A	1
I1LDX9	Uncharacterized protein OS=Glycine max OX=3847 GN=100805382 PE=4 SV=1	N/A	2	N/A	2	N/A	3
C6SYB1	Uncharacterized protein OS=Glycine max OX=3847 GN=100499931 PE=2 SV=1	N/A	1	N/A	1	N/A	1
I1KQ33	Uncharacterized protein OS=Glycine max OX=3847 GN=100814585 PE=4 SV=2	N/A	1	N/A	1	N/A	1
I1L8G3	Succinate--CoA ligase [ADP-forming] subunit beta, mitochondrial OS=Glycine max	N/A	1	N/A	1	N/A	1
C6T9D7	Uncharacterized protein OS=Glycine max OX=3847 GN=100803483 PE=2 SV=1	N/A	1	N/A	1	N/A	1
A0A0R0LAF9	Proteasome subunit alpha type OS=Glycine max OX=3847 GN=102662733 PE=3 SV=1	N/A	1	N/A	1	N/A	1

I1NIE7	T-complex protein 1 subunit gamma OS=Glycine max OX=3847 GN=100778141 PE=3 SV=2	N/A	1	N/A	1	N/A	1
C6TH59	Proteasome subunit alpha type OS=Glycine max OX=3847 GN=100799108 PE=2 SV=1	N/A	1	N/A	1	N/A	1
I1MH66	Xyloglucan endotransglucosylase/hydrolase OS=Glycine max OX=3847 GN=GLYMA_	N/A	1	N/A	1	N/A	1
C6SX86	Uncharacterized protein OS=Glycine max OX=3847 GN=100305936 PE=2 SV=1	N/A	1	N/A	1	N/A	1
A0A0R0GC70	Uncharacterized protein OS=Glycine max OX=3847 GN=100795017 PE=3 SV=1	N/A	1	N/A	1	N/A	1
A0A0R0F5U2	Uncharacterized protein OS=Glycine max OX=3847 GN=100782753 PE=4 SV=1	N/A	2	N/A	1	N/A	4
I1MJH1	Isocitrate dehydrogenase [NADP] OS=Glycine max OX=3847 GN=100816688 PE=3 SV=1	N/A	1	N/A	1	N/A	1
A0A0R0EXW6	Uncharacterized protein OS=Glycine max OX=3847 GN=100776168 PE=3 SV=1	N/A	2	N/A	2	N/A	3
A0A0R0JI98	Uncharacterized protein OS=Glycine max OX=3847 GN=100777627 PE=3 SV=1	N/A	1	N/A	1	N/A	1
A0A0R0GJV8	Uncharacterized protein OS=Glycine max OX=3847 GN=GLYMA_14G139600 PE=4 SV=1	N/A	1	N/A	1	N/A	1
K7LLZ6	Uncharacterized protein OS=Glycine max OX=3847 GN=100805723 PE=4 SV=1	N/A	1	N/A	1	N/A	14
I1KQ42	Uncharacterized protein OS=Glycine max OX=3847 GN=100813160 PE=3 SV=1	N/A	1	N/A	1	N/A	1
C6SW24	Uncharacterized protein OS=Glycine max OX=3847 GN=100499738 PE=2 SV=1	N/A	1	N/A	1	N/A	1
I1L056	Uncharacterized protein OS=Glycine max OX=3847 GN=100803933 PE=4 SV=2	N/A	1	N/A	1	N/A	1
I1MUR2	Uncharacterized protein OS=Glycine max OX=3847 GN=100778423 PE=3 SV=1	N/A	2	N/A	2	N/A	2
I1N8V4	Uncharacterized protein OS=Glycine max OX=3847 GN=100816616 PE=4 SV=1	N/A	1	N/A	1	N/A	1
I1JPW5	Uncharacterized protein OS=Glycine max OX=3847 GN=100811084 PE=3 SV=1	N/A	3	N/A	1	N/A	10
A0A0R0ESZ8	Elongation factor 1-alpha OS=Glycine max OX=3847 GN=100785429 PE=3 SV=1	N/A	3	N/A	3	N/A	9
I1M4L7	Beta-galactosidase OS=Glycine max OX=3847 GN=100804036 PE=3 SV=1	N/A	2	N/A	1	N/A	5
I1KEA0	Uncharacterized protein OS=Glycine max OX=3847 GN=100779078 PE=4 SV=1	N/A	3	N/A	3	N/A	5
P51061	Phosphoenolpyruvate carboxylase OS=Glycine max OX=3847 GN=PPC1 PE=2 SV=1	N/A	4	N/A	2	N/A	4
A0A0R0K474	Uncharacterized protein OS=Glycine max OX=3847 GN=100806638 PE=3 SV=1	N/A	3	N/A	3	N/A	4
I1KWM7	6-phosphogluconate dehydrogenase, decarboxylating	N/A	2	N/A	1	N/A	3
I1JHE9	Clathrin heavy chain OS=Glycine max OX=3847 GN=100808189 PE=3 SV=1	N/A	1	N/A	1	N/A	1
C6TM07	Uncharacterized protein OS=Glycine max OX=3847 GN=100810759 PE=2 SV=1	N/A	1	N/A	1	N/A	1
I1L0U6	Importin subunit alpha OS=Glycine max OX=3847 GN=100780020 PE=3 SV=1	N/A	1	N/A	1	N/A	1
A0A0R4J656	Uncharacterized protein OS=Glycine max OX=3847 GN=100811995 PE=3 SV=1	N/A	1	N/A	1	N/A	1
K7KQP0	Uncharacterized protein OS=Glycine max OX=3847 GN=100779210 PE=4 SV=1	N/A	1	N/A	1	N/A	1
A0A0R0KP31	Uncharacterized protein OS=Glycine max OX=3847 GN=100786325 PE=3 SV=1	N/A	1	N/A	1	N/A	1
I1JEH5	Uncharacterized protein OS=Glycine max OX=3847 GN=100809447 PE=4 SV=1	N/A	1	N/A	1	N/A	1
A0A0R0HF31	Uncharacterized protein OS=Glycine max OX=3847 GN=100801348 PE=3 SV=1	N/A	2	N/A	1	N/A	2
I1K5U0	Uncharacterized protein OS=Glycine max OX=3847 GN=GLYMA_05G231900 PE=3 SV=1	N/A	1	N/A	1	N/A	1
I1LKV9	Arogenate dehydratase OS=Glycine max OX=3847 GN=100800074 PE=4 SV=1	N/A	1	N/A	1	N/A	1
I1JR33	Glucose-6-phosphate 1-dehydrogenase OS=Glycine max OX=3847 GN=100811075 PE=3 SV=1	N/A	1	N/A	1	N/A	1
C6TJ60	Uncharacterized protein OS=Glycine max OX=3847 GN=100789547 PE=2 SV=1	N/A	1	N/A	1	N/A	1
I1LIP3	Uncharacterized protein OS=Glycine max OX=3847 GN=100818914 PE=4 SV=1	N/A	1	N/A	1	N/A	1

I1JL02	Peroxidase OS=Glycine max OX=3847 GN=100799894 PE=3 SV=2	N/A	1	N/A	1	N/A	2
K7LBF4	Uncharacterized protein OS=Glycine max OX=3847 GN=100775219 PE=4 SV=1	N/A	1	N/A	1	N/A	1
C6T231	Uncharacterized protein OS=Glycine max OX=3847 GN=100500550 PE=2 SV=1	N/A	1	N/A	1	N/A	1
I1L905	Phenylalanine ammonia-lyase OS=Glycine max OX=3847 GN=GLYMA_10G058200 PE=3 SV=1	N/A	1	N/A	1	N/A	1
K7LHS3	Uncharacterized protein OS=Glycine max OX=3847 GN=100812673 PE=4 SV=1	N/A	1	N/A	1	N/A	1
I1M1P1	Uncharacterized protein OS=Glycine max OX=3847 GN=100816306 PE=3 SV=1	N/A	1	N/A	1	N/A	1
I1MCZ9	Uncharacterized protein OS=Glycine max OX=3847 GN=100776791 PE=3 SV=1	N/A	1	N/A	1	N/A	1
A0A0R0FDV1	4-hydroxy-4-methyl-2-oxoglutarate aldolase OS=Glycine max OX=3847	N/A	1	N/A	1	N/A	1
I1JHJ8	Uncharacterized protein OS=Glycine max OX=3847 GN=100810328 PE=4 SV=1	N/A	1	N/A	1	N/A	1
K7K5S3	Uncharacterized protein OS=Glycine max OX=3847 GN=100817659 PE=4 SV=1	N/A	1	N/A	1	N/A	1
A0A0R0JPK0	Uncharacterized protein OS=Glycine max OX=3847 GN=100778164 PE=4 SV=1	N/A	1	N/A	1	N/A	1
I1KGP0	Uncharacterized protein OS=Glycine max OX=3847 GN=GLYMA_07G020000 PE=4 SV=1	N/A	1	N/A	1	N/A	1
A0A0R0F4W4	Uncharacterized protein OS=Glycine max OX=3847 GN=GLYMA_18G279000 PE=4 SV=1	N/A	1	N/A	1	N/A	1
I1MYY9	Laccase OS=Glycine max OX=3847 GN=GLYMA_18G023600 PE=3 SV=1	N/A	1	N/A	1	N/A	1
I1NA53	Uncharacterized protein OS=Glycine max OX=3847 GN=100796712 PE=4 SV=1	N/A	1	N/A	1	N/A	1
A0A0R0G2K1	Uncharacterized protein OS=Glycine max OX=3847 GN=100813688 PE=4 SV=1	N/A	1	N/A	1	N/A	1

The protein ID was obtained through blasting against soybean protein database. "PSM" means peptide spectrum matching and was used to estimate the protein abundance. "N/A" means not assigned.

Supplementary Table S2. Proteins identified in the seeds of *X. aethiopica* from Nigeria

Protein ID	Description	Peptides (N1)	Peptides (N2)	Unique peptides (N1)	Unique peptides (N2)	PSMs (N1)	PSMs (N2)
A0A0R0I373	Uncharacterized protein OS=Glycine max OX=3847 GN=GLYMA_09G032900 PE=3 SV=1	1	1	1	1	12	1
I1NAI6	Peroxiredoxin OS=Glycine max OX=3847 GN=100783307 PE=3 SV=1	1	1	1	1	2	2
P27066	Ribulose bisphosphate carboxylase large chain	1	3	1	3	4	6
I1JRK8	Proteasome subunit beta OS=Glycine max OX=3847 GN=GLYMA_03G244700 PE=3 SV=1	1	1	1	1	2	3
A0A0R0I5Z8	Lactoylglutathione lyase OS=Glycine max OX=3847 GN=GLYMA_09G004300 PE=3 SV=1	1	1	1	1	3	2
I1KYF8	40S ribosomal protein S8 OS=Glycine max OX=3847 GN=100803006 PE=3 SV=1	1	1	1	1	1	3
I1JNC7	Uncharacterized protein OS=Glycine max OX=3847 GN=100803795 PE=4 SV=1	1	N/A	1	N/A	1	N/A
I1KWQ3	Uncharacterized protein OS=Glycine max OX=3847 GN=GLYMA_08G256600 PE=4 SV=1	1	1	1	1	2	2
K7M9W4	Uncharacterized protein OS=Glycine max OX=3847 GN=100780332 PE=4 SV=1	1	1	1	1	1	1
I1K1X5	Proteasome subunit alpha type OS=Glycine max OX=3847 GN=100820387 PE=3 SV=1	1	2	1	2	1	2
A0A0R4J2P7	Uncharacterized protein OS=Glycine max OX=3847 GN=100817464 PE=4 SV=1	1	2	1	2	2	3
I1JRI7	Superoxide dismutase [Cu-Zn]	1	1	1	1	1	1
A0A0R0FHA5	Uncharacterized protein OS=Glycine max OX=3847 GN=GLYMA_17G053800 PE=3 SV=1	1	N/A	1	N/A	1	N/A
B0M1A9	Peroxisomal 3-ketoacyl-CoA thiolase OS=Glycine max OX=3847 GN=100170734 PE=2 SV=1	1	1	1	1	2	2
C6TLW3	Uncharacterized protein OS=Glycine max OX=3847 GN=100809582 PE=2 SV=1	1	N/A	1	N/A	1	N/A
I1NES8	Uncharacterized protein OS=Glycine max OX=3847 GN=GLYMA_20G090200 PE=3 SV=1	1	1	1	1	3	4
I1JFW6	Uncharacterized protein OS=Glycine max OX=3847 GN=100803239 PE=3 SV=1	1	2	1	2	2	4
C6T4H4	Ribulose-phosphate 3-epimerase OS=Glycine max OX=3847 GN=100527490 PE=2 SV=1	1	N/A	1	N/A	2	N/A
I1LAT2	Uncharacterized protein OS=Glycine max OX=3847 GN=100803952 PE=4 SV=1	1	1	1	1	5	2
I1MSH9	Signal peptidase I OS=Glycine max OX=3847 GN=100797179 PE=3 SV=1	1	1	1	1	1	1
I1KC25	Acetolactate synthase OS=Glycine max OX=3847 GN=Ahas2 PE=3 SV=2	1	N/A	1	N/A	1	N/A
I1JUT9	Uncharacterized protein OS=Glycine max OX=3847 GN=100807708 PE=4 SV=1	1	N/A	1	N/A	1	N/A
I1K609	Uncharacterized protein OS=Glycine max OX=3847 GN=100787769 PE=3 SV=1	1	1	1	1	1	2
I1L5K0	Uncharacterized protein OS=Glycine max OX=3847 GN=100803927 PE=4 SV=1	1	1	1	1	1	2
K7MJN4	Uncharacterized protein OS=Glycine max OX=3847 GN=100785204 PE=4 SV=1	1	1	1	1	3	1
I1N9J8	Uncharacterized protein OS=Glycine max OX=3847 GN=100790567 PE=4 SV=1	1	1	1	1	2	2
I1K0W4	Glutamate dehydrogenase OS=Glycine max OX=3847 GN=100793231 PE=3 SV=1	1	1	1	1	2	1
I1LUL9	Ketol-acid reductoisomerase OS=Glycine max OX=3847 GN=100799921 PE=3 SV=1	1	1	1	1	2	1
K7M4G7	Uncharacterized protein OS=Glycine max OX=3847 GN=GLYMA_14G020500 PE=4 SV=1	1	1	1	1	2	1
I1JZW5	Uncharacterized protein OS=Glycine max OX=3847 GN=GLYMA_05G033500 PE=3 SV=1	1	1	1	1	1	1
B0M1B1	Peroxisomal glycolate oxidase OS=Glycine max OX=3847 GN=100777153 PE=2 SV=1	1	N/A	1	N/A	1	N/A
I1JKK8	Uncharacterized protein OS=Glycine max OX=3847 GN=100783833 PE=4 SV=2	1	N/A	1	N/A	1	N/A

C6SVB8	40S ribosomal protein S24 OS=Glycine max OX=3847 GN=100499673 PE=2 SV=1	1	1	1	1	1	1
I1J4G4	Profilin OS=Glycine max OX=3847 GN=100778045 PE=3 SV=1	1	1	1	1	3	2
I1M8A5	Proteasome subunit alpha type OS=Glycine max OX=3847 GN=100792921 PE=3 SV=1	1	1	1	1	2	1
I1JZ45	Uncharacterized protein OS=Glycine max OX=3847 GN=100802238 PE=3 SV=2	1	1	1	1	1	1
I1L8S1	Uncharacterized protein OS=Glycine max OX=3847 GN=100796694 PE=3 SV=1	1	1	1	1	1	1
A0A0R0J965	Uncharacterized protein OS=Glycine max OX=3847 GN=100809384 PE=4 SV=1	1	2	1	2	1	3
I1JXS7	Uncharacterized protein OS=Glycine max OX=3847 GN=100802236 PE=3 SV=1	1	2	1	2	2	2
I1J525	ATP-dependent 6-phosphofructokinase	1	2	1	2	1	2
I1KL36	Uncharacterized protein OS=Glycine max OX=3847 GN=GLYMA_07G180100 PE=3 SV=1	1	1	1	1	2	2
I1JWG8	Uncharacterized protein OS=Glycine max OX=3847 GN=GLYMA_04G140400 PE=4 SV=1	1	N/A	1	N/A	1	N/A
K7KZN7	Uncharacterized protein OS=Glycine max OX=3847 GN=GLYMA_07G046000 PE=3 SV=1	1	1	1	1	2	1
A0A0R0KPC8	Uncharacterized protein OS=Glycine max OX=3847 GN=GLYMA_03G246300 PE=3 SV=1	1	1	1	1	1	1
I1LV40	Uncharacterized protein OS=Glycine max OX=3847 GN=100799923 PE=4 SV=1	1	1	1	1	1	1
K7KWB0	Uncharacterized protein OS=Glycine max OX=3847 GN=100787451 PE=3 SV=1	1	N/A	1	N/A	1	N/A
I1KY16	Uncharacterized protein OS=Glycine max OX=3847 GN=100814236 PE=3 SV=1	1	1	1	1	2	2
B0M1A3	Peroxisomal hydroxypyruvate reductase OS=Glycine max OX=3847 GN=547640 PE=2 SV=1	1	1	1	1	1	1
C6SZW2	Uncharacterized protein OS=Glycine max OX=3847 GN=100805631 PE=2 SV=1	1	1	1	1	1	1
A0A0R0HBQ2	Uncharacterized protein OS=Glycine max OX=3847 GN=GLYMA_13G367000 PE=4 SV=1	1	1	1	1	1	1
I1LFG4	Uncharacterized protein OS=Glycine max OX=3847 GN=100781676 PE=4 SV=1	1	1	1	1	1	1
A0A0R0L5B0	Uncharacterized protein OS=Glycine max OX=3847 GN=GLYMA_02G165300 PE=4 SV=1	1	N/A	1	N/A	3	N/A
I1J8Q7	Uncharacterized protein OS=Glycine max OX=3847 GN=100787497 PE=4 SV=1	1	N/A	1	N/A	1	N/A
C6T078	Uncharacterized protein OS=Glycine max OX=3847 GN=100306624 PE=2 SV=1	1	1	1	1	1	1
I1M643	Uncharacterized protein OS=Glycine max OX=3847 GN=100790110 PE=4 SV=1	1	2	1	2	1	2
A0A0R4J3N1	Uncharacterized protein OS=Glycine max OX=3847 GN=100779787 PE=3 SV=1	1	N/A	1	N/A	1	N/A
C6TD38	Uncharacterized protein OS=Glycine max OX=3847 GN=100805113 PE=2 SV=1	1	N/A	1	N/A	1	N/A
A0A0R0FND9	Uncharacterized protein OS=Glycine max OX=3847 GN=100814972 PE=3 SV=1	1	1	1	1	2	2
A0A0R4J5H9	Xyloglucan endotransglucosylase/hydrolase	1	1	1	1	1	1
K7KBZ4	Cysteine synthase OS=Glycine max OX=3847 GN=GLYMA_03G006700 PE=3 SV=1	1	N/A	1	N/A	1	N/A
A0A0R0GEU1	Glucose-6-phosphate isomerase	1	2	1	2	2	2
I1K6N2	Uncharacterized protein OS=Glycine max OX=3847 GN=100805621 PE=4 SV=1	1	2	1	2	1	2
I1JD96	Uncharacterized protein OS=Glycine max OX=3847 GN=100778071 PE=4 SV=1	1	1	1	1	1	1
C6TGN8	Uncharacterized protein OS=Glycine max OX=3847 GN=100797023 PE=2 SV=1	1	1	1	1	2	2
I1JJ62	Citrate synthase OS=Glycine max OX=3847 GN=100784332 PE=3 SV=1	1	3	1	3	2	6
A0A0R0IY70	Serine/threonine-protein phosphatase 2A 55 kDa regulatory subunit B	1	N/A	1	N/A	1	N/A
C6SWA6	Histone H2A OS=Glycine max OX=3847 GN=100305705 PE=2 SV=1	1	1	1	1	3	2
I1JT85	Uncharacterized protein OS=Glycine max OX=3847 GN=100800985 PE=3 SV=1	1	N/A	1	N/A	2	N/A

I1JGB5	Eukaryotic translation initiation factor 3 subunit E	1	1	1	1	1	1
A0A0R0K0Z8	Uncharacterized protein OS=Glycine max OX=3847 GN=100784412 PE=4 SV=1	1	N/A	1	N/A	1	N/A
K7MXZ0	GrpE protein homolog OS=Glycine max OX=3847 GN=100820521 PE=3 SV=1	1	N/A	1	N/A	1	N/A
A0A0R0ES33	Uncharacterized protein OS=Glycine max OX=3847 GN=GLYMA_19G237000 PE=4 SV=1	1	N/A	1	N/A	1	N/A
A0A0R0F137	Uncharacterized protein OS=Glycine max OX=3847 GN=GLYMA_19G232100 PE=4 SV=1	1	1	1	1	2	2
I1KH71	Acetyltransferase component of pyruvate dehydrogenase complex	1	1	1	1	2	1
C6TK46	Uncharacterized protein OS=Glycine max OX=3847 GN=GLYMA_08G312400 PE=2 SV=1	1	N/A	1	N/A	1	N/A
K7LTG4	Uncharacterized protein OS=Glycine max OX=3847 GN=100784046 PE=3 SV=1	1	N/A	1	N/A	1	N/A
C6TN61	Uncharacterized protein OS=Glycine max OX=3847 GN=100787298 PE=2 SV=1	1	1	1	1	2	1
I1N6R2	60S ribosomal protein L36 OS=Glycine max OX=3847 GN=100526979 PE=3 SV=1	1	1	1	1	3	1
A0A0R0EWX9	Uncharacterized protein OS=Glycine max OX=3847 GN=100791967 PE=4 SV=1	1	1	1	1	3	2
I1LVQ6	Ribulose bisphosphate carboxylase small chain	1	1	1	1	2	2
I1JXH8	Uncharacterized protein OS=Glycine max OX=3847 GN=100776903 PE=3 SV=1	1	N/A	1	N/A	1	N/A
I1M322	Uncharacterized protein OS=Glycine max OX=3847 GN=100807226 PE=4 SV=2	1	1	1	1	2	1
I1K5Z4	Phosphotransferase OS=Glycine max OX=3847 GN=100783175 PE=3 SV=1	1	1	1	1	1	1
I1N7G4	Uncharacterized protein OS=Glycine max OX=3847 GN=100808230 PE=3 SV=1	1	N/A	1	N/A	1	N/A
I1NF03	Uncharacterized protein OS=Glycine max OX=3847 GN=100811997 PE=3 SV=1	1	2	1	2	1	2
A0A0R0JKN5	Uncharacterized protein OS=Glycine max OX=3847 GN=100815571 PE=4 SV=1	1	1	1	1	1	1
I1J9W0	40S ribosomal protein S30 OS=Glycine max OX=3847 GN=GLYMA_01G209400 PE=3 SV=2	1	1	1	1	1	1
A0A0R0HSV1	Uncharacterized protein OS=Glycine max OX=3847 GN=100800747 PE=3 SV=1	1	1	1	1	1	1
I1ME62	4-hydroxy-4-methyl-2-oxoglutarate aldolase	1	1	1	1	1	1
I1MQT7	Uncharacterized protein OS=Glycine max OX=3847 GN=100816200 PE=4 SV=2	1	1	1	1	1	1
K7L1B9	Uncharacterized protein OS=Glycine max OX=3847 GN=GLYMA_07G108800 PE=4 SV=1	1	1	1	1	2	2
I1MU40	Uncharacterized protein OS=Glycine max OX=3847 GN=100798787 PE=3 SV=1	1	N/A	1	N/A	1	N/A
K7K5P1	Uncharacterized protein OS=Glycine max OX=3847 GN=GLYMA_01G245700 PE=4 SV=1	1	N/A	1	N/A	1	N/A
A0A0R4J3P8	Uncharacterized protein OS=Glycine max OX=3847 GN=100787986 PE=4 SV=1	1	1	1	1	2	1
I1LF17	Uncharacterized protein OS=Glycine max OX=3847 GN=100782932 PE=4 SV=1	1	N/A	1	N/A	1	N/A
I1LTF6	Uncharacterized protein OS=Glycine max OX=3847 GN=100809324 PE=3 SV=1	1	N/A	1	N/A	1	N/A
I1KQJ6	Eukaryotic translation initiation factor 3 subunit C	1	N/A	1	N/A	1	N/A
I1LIG5	Uncharacterized protein OS=Glycine max OX=3847 GN=100797423 PE=4 SV=1	1	N/A	1	N/A	1	N/A
A0A0R4J2P9	Cysteine synthase OS=Glycine max OX=3847 GN=100815330 PE=3 SV=1	1	1	1	1	1	1
I1M9V9	Uncharacterized protein OS=Glycine max OX=3847 GN=100306164 PE=4 SV=2	1	N/A	1	N/A	1	N/A
Q8W1A0	Cysteine synthase OS=Glycine max OX=3847 GN=547631 PE=2 SV=1	1	2	1	2	1	2
I1LBD0	Uncharacterized protein OS=Glycine max OX=3847 GN=100806082 PE=4 SV=1	1	N/A	1	N/A	1	N/A
A0A0R4J5B9	Peroxiredoxin OS=Glycine max OX=3847 GN=GLYMA_15G105600 PE=3 SV=1	1	1	1	1	1	1
I1JG72	Uncharacterized protein OS=Glycine max OX=3847 GN=100805704 PE=4 SV=1	1	1	1	1	1	1

I1K743	Uncharacterized protein OS=Glycine max OX=3847 GN=GLYMA_06G009700 PE=3 SV=2	1	N/A	1	N/A	1	N/A
I1MR04	Uncharacterized protein OS=Glycine max OX=3847 GN=GLYMA_17G008100 PE=4 SV=1	1	N/A	1	N/A	1	N/A
I1N3Z3	Uncharacterized protein OS=Glycine max OX=3847 GN=GLYMA_18G242300 PE=4 SV=2	1	N/A	1	N/A	2	N/A
I1LP98	Uncharacterized protein OS=Glycine max OX=3847 GN=100790764 PE=4 SV=1	1	1	1	1	1	2
I1JVU2	60S ribosomal protein L13 OS=Glycine max OX=3847 GN=100805957 PE=3 SV=1	1	1	1	1	1	1
A0A0R0LCZ4	Uncharacterized protein OS=Glycine max OX=3847 GN=100811217 PE=3 SV=1	1	1	1	1	2	1
I1MSU1	Xylose isomerase OS=Glycine max OX=3847 GN=100804624 PE=3 SV=1	1	1	1	1	1	2
I1JGN1	Uncharacterized protein OS=Glycine max OX=3847 GN=100780041 PE=3 SV=1	1	1	1	1	1	2
A0A0R0L372	GrpE protein homolog OS=Glycine max OX=3847 GN=100803396 PE=3 SV=1	1	1	1	1	1	1
I1L386	Uncharacterized protein OS=Glycine max OX=3847 GN=100780914 PE=3 SV=1	1	N/A	1	N/A	1	N/A
I1LZJ8	Uncharacterized protein OS=Glycine max OX=3847 GN=100791477 PE=3 SV=1	1	1	1	1	1	1
I1J550	Uncharacterized protein OS=Glycine max OX=3847 GN=100799836 PE=4 SV=1	1	1	1	1	2	2
A0A0R0E8K1	Uncharacterized protein OS=Glycine max OX=3847 GN=100796915 PE=4 SV=1	1	1	1	1	1	1
I1J717	Proteasome subunit alpha type OS=Glycine max OX=3847 GN=100807110 PE=3 SV=1	1	N/A	1	N/A	1	N/A
I1LDX9	Uncharacterized protein OS=Glycine max OX=3847 GN=100805382 PE=4 SV=1	1	N/A	1	N/A	1	N/A
A0A0R0HYJ0	Uncharacterized protein OS=Glycine max OX=3847 GN=100803600 PE=4 SV=1	1	2	1	2	1	2
I1LMN0	Trafficking protein particle complex subunit	1	N/A	1	N/A	1	N/A
I1M4H1	Carboxypeptidase OS=Glycine max OX=3847 GN=100797295 PE=3 SV=1	1	1	1	1	1	1
I1J4X9	Uncharacterized protein OS=Glycine max OX=3847 GN=100776634 PE=3 SV=1	1	N/A	1	N/A	1	N/A
C6TMX6	Cysteine synthase OS=Glycine max OX=3847 GN=100101865 PE=2 SV=1	1	1	1	1	1	2
C6SYB1	Uncharacterized protein OS=Glycine max OX=3847 GN=100499931 PE=2 SV=1	1	N/A	1	N/A	1	N/A
I1KQ33	Uncharacterized protein OS=Glycine max OX=3847 GN=100814585 PE=4 SV=2	1	N/A	1	N/A	1	N/A
A0A0R0KVT1	Uncharacterized protein OS=Glycine max OX=3847 GN=GLYMA_03G172700 PE=3 SV=1	1	1	1	1	1	2
A0A0R0HNI5	Acyl-coenzyme A oxidase OS=Glycine max OX=3847 GN=GLYMA_11G035200 PE=3 SV=1	1	N/A	1	N/A	1	N/A
I1KG56	Coatomer subunit beta OS=Glycine max OX=3847 GN=100802469 PE=4 SV=1	1	1	1	1	1	1
I1JLY5	Uncharacterized protein OS=Glycine max OX=3847 GN=GLYMA_04G107900 PE=4 SV=1	1	1	1	1	1	1
I1L8G3	Succinate--CoA ligase [ADP-forming] subunit beta, mitochondrial	1	2	1	2	2	3
C6T9D7	Uncharacterized protein OS=Glycine max OX=3847 GN=100803483 PE=2 SV=1	1	1	1	1	1	2
A0A0R4J4L8	Uncharacterized protein OS=Glycine max OX=3847 GN=100819819 PE=3 SV=1	1	2	1	1	1	2
K7L085	Uncharacterized protein OS=Glycine max OX=3847 GN=100791174 PE=4 SV=1	1	N/A	1	N/A	1	N/A
A0A0R4J559	Uncharacterized protein OS=Glycine max OX=3847 GN=100789057 PE=4 SV=1	1	N/A	1	N/A	1	N/A
C6SWV2	V-type proton ATPase subunit G OS=Glycine max OX=3847 GN=100499803 PE=2 SV=1	1	N/A	1	N/A	1	N/A
K7KTV2	Uncharacterized protein OS=Glycine max OX=3847 GN=100811861 PE=4 SV=1	1	1	1	1	1	1
I1LJY0	Uncharacterized protein OS=Glycine max OX=3847 GN=100799534 PE=4 SV=1	1	N/A	1	N/A	1	N/A
I1JVL2	Uncharacterized protein OS=Glycine max OX=3847 GN=100788283 PE=3 SV=1	1	N/A	1	N/A	4	N/A
A0A0R0HSJ5	Peptidyl-prolyl cis-trans isomerase	1	N/A	1	N/A	2	N/A

I1LEK6	Uncharacterized protein OS=Glycine max OX=3847 GN=100781483 PE=4 SV=1	1	1	1	1	1	1
C6SVV1	Uncharacterized protein OS=Glycine max OX=3847 GN=100499720 PE=2 SV=1	1	N/A	1	N/A	2	N/A
A0A0R0HJE4	Uncharacterized protein OS=Glycine max OX=3847 GN=GLYMA_11G194200 PE=4 SV=1	1	1	1	1	2	2
K7KHP9	Uncharacterized protein OS=Glycine max OX=3847 GN=100775279 PE=4 SV=1	1	N/A	1	N/A	1	N/A
I1LVH8	Peroxidase OS=Glycine max OX=3847 GN=100796737 PE=3 SV=1	1	1	1	1	1	1
I1L7D4	Uncharacterized protein OS=Glycine max OX=3847 GN=100793713 PE=3 SV=1	1	1	1	1	1	1
I1L1H2	Uncharacterized protein OS=Glycine max OX=3847 GN=100779302 PE=4 SV=1	1	N/A	1	N/A	1	N/A
A0A0R0LAF9	Proteasome subunit alpha type OS=Glycine max OX=3847 GN=102662733 PE=3 SV=1	1	1	1	1	1	1
I1KPM3	Uncharacterized protein OS=Glycine max OX=3847 GN=100798044 PE=3 SV=1	1	N/A	1	N/A	1	N/A
A0A0R0ELF0	Uncharacterized protein OS=Glycine max OX=3847 GN=100817706 PE=4 SV=1	1	N/A	1	N/A	1	N/A
I1KBX4	Alpha-mannosidase OS=Glycine max OX=3847 GN=100802094 PE=3 SV=2	1	N/A	1	N/A	1	N/A
I1J693	Uncharacterized protein OS=Glycine max OX=3847 GN=100797542 PE=4 SV=1	1	N/A	1	N/A	1	N/A
F6KBT3	Allene oxide cyclase 3 OS=Glycine max OX=3847 GN=AOC3 PE=2 SV=1	1	N/A	1	N/A	1	N/A
I1M8K6	Uncharacterized protein OS=Glycine max OX=3847 GN=100778174 PE=4 SV=1	1	1	1	1	1	1
I1LGM2	Uncharacterized protein OS=Glycine max OX=3847 GN=100816776 PE=4 SV=1	1	N/A	1	N/A	2	N/A
I1NHJ6	Peptidyl-prolyl cis-trans isomerase OS=Glycine max OX=3847 GN=100781538 PE=3 SV=1	1	N/A	1	N/A	1	N/A
I1LJ68	Fructose-bisphosphate aldolase OS=Glycine max OX=3847 GN=100818915 PE=3 SV=1	1	N/A	1	N/A	1	N/A
A0A0R4J5X9	Succinate dehydrogenase [ubiquinone] iron-sulfur subunit, mitochondrial	1	1	1	1	1	1
I1KP02	Uncharacterized protein OS=Glycine max OX=3847 GN=100788197 PE=4 SV=2	1	N/A	1	N/A	1	N/A
I1N5S0	Formate dehydrogenase, mitochondrial	1	N/A	1	N/A	1	N/A
I1KUN0	Uncharacterized protein OS=Glycine max OX=3847 GN=100797897 PE=4 SV=2	1	1	1	1	1	1
A0A368UHK6	Uncharacterized protein OS=Glycine max OX=3847 GN=GLYMA_10G123500 PE=3 SV=1	1	1	1	1	1	1
C6TAN4	Uncharacterized protein OS=Glycine max OX=3847 GN=100784099 PE=2 SV=1	1	N/A	1	N/A	1	N/A
C6TIQ8	Uncharacterized protein OS=Glycine max OX=3847 GN=100788470 PE=2 SV=1	1	N/A	1	N/A	1	N/A
I1KKN7	Pyrophosphate--fructose 6-phosphate 1-phosphotransferase subunit alpha	1	N/A	1	N/A	1	N/A
I1LIK8	Uncharacterized protein OS=Glycine max OX=3847 GN=100809292 PE=3 SV=2	1	N/A	1	N/A	1	N/A
I1LXQ1	Uncharacterized protein OS=Glycine max OX=3847 GN=100802266 PE=4 SV=2	1	2	1	2	1	2
C6TJH2	Uncharacterized protein OS=Glycine max OX=3847 GN=100810611 PE=2 SV=1	1	1	1	1	1	1
C6SWX1	Uncharacterized protein OS=Glycine max OX=3847 GN=100499810 PE=2 SV=1	1	N/A	1	N/A	3	N/A
I1NIE7	T-complex protein 1 subunit gamma OS=Glycine max OX=3847 GN=100778141 PE=3 SV=2	1	1	1	1	1	1
I1MU00	Uncharacterized protein OS=Glycine max OX=3847 GN=100784140 PE=3 SV=1	1	1	1	1	2	2
C6SVR8	Uncharacterized protein OS=Glycine max OX=3847 GN=100305587 PE=2 SV=1	1	N/A	1	N/A	1	N/A
I1JYG7	Uncharacterized protein OS=Glycine max OX=3847 GN=GLYMA_04G227400 PE=4 SV=1	1	N/A	1	N/A	1	N/A
I1L8R1	Uncharacterized protein OS=Glycine max OX=3847 GN=100786828 PE=4 SV=1	1	1	1	1	1	1
Q9SDZ0	Isoflavone reductase homolog 2 OS=Glycine max OX=3847 GN=IFR2 PE=2 SV=1	1	N/A	1	N/A	1	N/A
I1MAL3	Ubiquitin-fold modifier 1 OS=Glycine max OX=3847 GN=100815619 PE=3 SV=1	1	N/A	1	N/A	1	N/A

I1K3R6	Uncharacterized protein OS=Glycine max OX=3847 GN=100813286 PE=4 SV=1	1	1	1	1	1	1
I1LMS5	Ribosomal protein OS=Glycine max OX=3847 GN=100499715 PE=3 SV=1	1	1	1	1	1	1
I1JMQ0	Nucleoside diphosphate kinase	1	1	1	1	2	2
A0A0R0I9G1	Uncharacterized protein OS=Glycine max OX=3847 GN=100780375 PE=3 SV=1	1	N/A	1	N/A	1	N/A
I1JZ89	Uncharacterized protein OS=Glycine max OX=3847 GN=100814682 PE=4 SV=1	1	1	1	1	1	1
I1LII4	Uncharacterized protein OS=Glycine max OX=3847 GN=100802384 PE=4 SV=2	1	N/A	1	N/A	1	N/A
K7KTL1	Uncharacterized protein OS=Glycine max OX=3847 GN=GLYMA_06G072300 PE=4 SV=1	1	N/A	1	N/A	1	N/A
I1KRU1	Uncharacterized protein OS=Glycine max OX=3847 GN=100798407 PE=4 SV=1	1	N/A	1	N/A	1	N/A
I1LKR1	Uncharacterized protein OS=Glycine max OX=3847 GN=100784551 PE=4 SV=1	1	N/A	1	N/A	1	N/A
A0A0R0JZ02	Uncharacterized protein OS=Glycine max OX=3847 GN=GLYMA_05G087200 PE=4 SV=1	1	N/A	1	N/A	1	N/A
I1KEL5	Uncharacterized protein OS=Glycine max OX=3847 GN=100800847 PE=4 SV=1	1	N/A	1	N/A	1	N/A
I1JMB0	Protein transport protein SEC23 OS=Glycine max OX=3847 GN=100803793 PE=3 SV=1	1	N/A	1	N/A	1	N/A
A0A0R0FW44	Uncharacterized protein OS=Glycine max OX=3847 GN=100805853 PE=3 SV=1	1	1	1	1	1	1
I1K2F3	Uncharacterized protein OS=Glycine max OX=3847 GN=100782286 PE=4 SV=1	1	1	1	1	1	1
A0A0R0K544	GTP-binding nuclear protein OS=Glycine max OX=3847 GN=100814146 PE=3 SV=1	1	3	1	3	1	3
I1JAU2	Uncharacterized protein OS=Glycine max OX=3847 GN=100809773 PE=3 SV=1	1	1	1	1	1	1
A0A0R0FG80	Uncharacterized protein OS=Glycine max OX=3847 GN=100782718 PE=4 SV=1	1	N/A	1	N/A	1	N/A
A0A0R0H2H7	Uncharacterized protein OS=Glycine max OX=3847 GN=GLYMA_12G071600 PE=4 SV=1	1	1	1	1	1	5
I1MEK2	Uncharacterized protein OS=Glycine max OX=3847 GN=GLYMA_15G075300 PE=4 SV=1	1	N/A	1	N/A	1	N/A
A0A0R0I282	Uncharacterized protein OS=Glycine max OX=3847 GN=GLYMA_10G160100 PE=4 SV=1	1	N/A	1	N/A	1	N/A
A0A368UK73	Alanine--tRNA ligase OS=Glycine max OX=3847 GN=GLYMA_11G102100 PE=3 SV=1	1	1	1	1	1	1
C6T5U0	Uncharacterized protein OS=Glycine max OX=3847 GN=100527911 PE=2 SV=1	1	2	1	2	1	2
A0A0R0F259	Uncharacterized protein OS=Glycine max OX=3847 GN=100782747 PE=3 SV=1	1	N/A	1	N/A	1	N/A
K7M4P2	Uncharacterized protein OS=Glycine max OX=3847 GN=GLYMA_14G030900 PE=4 SV=1	1	1	1	1	2	2
A0A0R0JKX7	Uncharacterized protein OS=Glycine max OX=3847 GN=100796232 PE=4 SV=1	1	N/A	1	N/A	1	N/A
C6TH59	Proteasome subunit alpha type OS=Glycine max OX=3847 GN=100799108 PE=2 SV=1	1	2	1	2	1	2
P02957	Photosystem II protein D1 OS=Glycine max OX=3847 GN=psbA PE=3 SV=3	1	1	1	1	1	1
I1KJ91	Uncharacterized protein OS=Glycine max OX=3847 GN=100777314 PE=4 SV=1	1	N/A	1	N/A	1	N/A
I1JD34	Uncharacterized protein OS=Glycine max OX=3847 GN=100804818 PE=3 SV=1	1	N/A	1	N/A	1	N/A
C6SV97	Peptidyl-prolyl cis-trans isomerase OS=Glycine max OX=3847 GN=100305485 PE=2 SV=1	1	N/A	1	N/A	1	N/A
K7LWP1	Uncharacterized protein OS=Glycine max OX=3847 GN=100792174 PE=3 SV=1	1	N/A	1	N/A	2	N/A
K7MBV6	Uncharacterized protein OS=Glycine max OX=3847 GN=GLYMA_15G167400 PE=4 SV=1	1	1	1	1	1	1
I1LBX9	Uncharacterized protein OS=Glycine max OX=3847 GN=100809275 PE=4 SV=2	1	N/A	1	N/A	1	N/A
I1MG91	Uncharacterized protein OS=Glycine max OX=3847 GN=100781784 PE=4 SV=1	1	N/A	1	N/A	1	N/A
I1N0W4	Xyloglucan endotransglucosylase/hydrolase OS=Glycine max OX=3847	1	1	1	1	3	4
C6SV94	Uncharacterized protein OS=Glycine max OX=3847 GN=100786325 PE=2 SV=1	1	1	1	1	1	1

I1KT48	Uncharacterized protein OS=Glycine max OX=3847 GN=100819908 PE=4 SV=1	1	N/A	1	N/A	1	N/A
I1LSW3	Uncharacterized protein OS=Glycine max OX=3847 GN=100801346 PE=4 SV=1	1	N/A	1	N/A	1	N/A
A0A0R0IAF2	Peroxiredoxin OS=Glycine max OX=3847 GN=GLYMA_09G192800 PE=3 SV=1	1	1	1	1	3	2
K7MIW7	Uncharacterized protein OS=Glycine max OX=3847 GN=100816018 PE=4 SV=1	1	N/A	1	N/A	1	N/A
Q42797	Trans-cinnamate 4-monooxygenase OS=Glycine max OX=3847 GN=CYP73A11 PE=2 SV=1	1	1	1	1	1	1
A0A0R0EVM7	Glutamine synthetase OS=Glycine max OX=3847 GN=GLYMA_18G041100 PE=3 SV=1	1	1	1	1	1	1
I1KYU6	Alpha-1,4 glucan phosphorylase OS=Glycine max OX=3847 GN=100789789 PE=3 SV=1	1	N/A	1	N/A	1	N/A
I1N8I9	Uncharacterized protein OS=Glycine max OX=3847 GN=100803801 PE=4 SV=1	1	N/A	1	N/A	1	N/A
I1MH66	Xyloglucan endotransglucosylase/hydrolase OS=Glycine max	1	N/A	1	N/A	1	N/A
I1JRA0	Uncharacterized protein OS=Glycine max OX=3847 GN=100807867 PE=3 SV=1	1	N/A	1	N/A	1	N/A
K7M7D7	Uncharacterized protein OS=Glycine max OX=3847 GN=GLYMA_14G162000 PE=4 SV=1	1	N/A	1	N/A	1	N/A
A0A0R0K2I7	Uncharacterized protein OS=Glycine max OX=3847 GN=100781347 PE=4 SV=1	1	N/A	1	N/A	1	N/A
C6SX86	Uncharacterized protein OS=Glycine max OX=3847 GN=100305936 PE=2 SV=1	1	N/A	1	N/A	1	N/A
I1JTW0	Uncharacterized protein OS=Glycine max OX=3847 GN=100817153 PE=3 SV=1	1	N/A	1	N/A	1	N/A
I1L7U8	Uncharacterized protein OS=Glycine max OX=3847 GN=100792839 PE=3 SV=1	1	N/A	1	N/A	1	N/A
K7LGT6	Uncharacterized protein OS=Glycine max OX=3847 GN=GLYMA_10G010900 PE=4 SV=1	1	N/A	1	N/A	1	N/A
I1K499	Transmembrane 9 superfamily member OS=Glycine max OX=3847	1	N/A	1	N/A	1	N/A
A0A0R0KHB4	Delta-aminolevulinic acid dehydratase OS=Glycine max OX=3847	1	2	1	2	1	2
K7K760	RNA-dependent RNA polymerase OS=Glycine max OX=3847 GN=100811587 PE=3 SV=1	1	N/A	1	N/A	1	N/A
I1L3Z2	Glutamate decarboxylase OS=Glycine max OX=3847 GN=100796475 PE=3 SV=1	1	N/A	1	N/A	1	N/A
A0A0R0HR30	Uncharacterized protein OS=Glycine max OX=3847 GN=100500084 PE=4 SV=1	1	1	1	1	1	1
I1MAW4	Uncharacterized protein OS=Glycine max OX=3847 GN=100794661 PE=4 SV=1	1	N/A	1	N/A	1	N/A
K7LPA3	Uncharacterized protein OS=Glycine max OX=3847 GN=GLYMA_11G118700 PE=4 SV=1	1	N/A	1	N/A	1	N/A
I1K9A3	Uncharacterized protein OS=Glycine max OX=3847 GN=100788146 PE=4 SV=1	1	N/A	1	N/A	1	N/A
A0A0R0GC70	Uncharacterized protein OS=Glycine max OX=3847 GN=100795017 PE=3 SV=1	1	1	1	1	1	1
I1MZW3	Uncharacterized protein OS=Glycine max OX=3847 GN=100780392 PE=3 SV=2	1	N/A	1	N/A	1	N/A
I1KRJ0	6,7-dimethyl-8-ribityllumazine synthase OS=Glycine max OX=3847	1	N/A	1	N/A	1	N/A
A0A0R0KIG7	Uncharacterized protein (Fragment) OS=Glycine max OX=3847	1	N/A	1	N/A	1	N/A
A0A0R4J2U8	Uncharacterized protein OS=Glycine max OX=3847 GN=100818028 PE=4 SV=1	1	N/A	1	N/A	1	N/A
A0A0R0FC01	tRNA (guanine(37)-N1)-methyltransferase OS=Glycine max	1	N/A	1	N/A	1	N/A
I1JM15	Uncharacterized protein OS=Glycine max OX=3847 GN=100779530 PE=4 SV=1	1	N/A	1	N/A	1	N/A
I1KM82	Uncharacterized protein OS=Glycine max OX=3847 GN=GLYMA_07G221200 PE=4 SV=1	1	N/A	1	N/A	1	N/A
E3NYP7	Mother of flowering locus T-like protein OS=Glycine max OX=3847 GN=MFTL PE=2 SV=1	1	1	1	1	2	3
K7MYN7	Uncharacterized protein OS=Glycine max OX=3847 GN=102660083 PE=4 SV=1	1	N/A	1	N/A	1	N/A
I1K7A7	Glyceraldehyde-3-phosphate dehydrogenase OS=Glycine max OX=3847 GN=GLYMA_06G015900 PE=3 SV=1	1	N/A	1	N/A	1	N/A
K7M387	Uncharacterized protein OS=Glycine max OX=3847 GN=100819867 PE=3 SV=1	1	N/A	1	N/A	1	N/A

K7KDQ9	Uncharacterized protein OS=Glycine max OX=3847 GN=100814846 PE=4 SV=1	1	N/A	1	N/A	1	N/A
K7MZE3	Uncharacterized protein OS=Glycine max OX=3847 GN=102668708 PE=4 SV=1	1	1	1	1	1	1
I1MCZ2	Protein ROOT HAIR DEFECTIVE 3 homolog OS=Glycine max	1	N/A	1	N/A	1	N/A
A0A0R0K6F3	Uncharacterized protein OS=Glycine max OX=3847 GN=100800789 PE=4 SV=1	1	N/A	1	N/A	1	N/A
I1LZ93	Isocitrate dehydrogenase [NAD] subunit, mitochondrial	2	2	2	2	5	2
I1KTX8	Phosphoglycerate kinase OS=Glycine max OX=3847 GN=100795933 PE=3 SV=1	2	N/A	2	N/A	7	N/A
I1MBR7	UTP--glucose-1-phosphate uridylyltransferase OS=Glycine max	2	3	2	3	9	9
A0A0R0HEQ3	Histone H2B OS=Glycine max OX=3847 GN=100778668 PE=3 SV=1	2	3	2	3	4	4
I1MQS6	EF1Bgamma class glutathione S-transferase OS=Glycine max	2	2	2	2	4	5
A0A0R0EXX1	Pyruvate kinase OS=Glycine max OX=3847 GN=100781158 PE=3 SV=1	2	2	2	2	3	3
A0A0R4J2M8	Proteasome subunit alpha type OS=Glycine max OX=3847 GN=100808731 PE=3 SV=1	2	4	2	4	4	8
I1LMP2	Uncharacterized protein OS=Glycine max OX=3847 GN=100790393 PE=4 SV=1	2	1	2	1	2	2
I1JXI9	Proteasome subunit beta OS=Glycine max OX=3847 GN=100778498 PE=3 SV=1	2	2	2	2	4	3
I1K5G1	T-complex protein 1 subunit delta OS=Glycine max OX=3847 GN=100786888 PE=3 SV=1	2	1	2	1	3	1
A0A0R0KN50	Uncharacterized protein OS=Glycine max OX=3847 GN=GLYMA_03G222100 PE=3 SV=1	2	2	2	2	2	2
I1MGE7	Uncharacterized protein OS=Glycine max OX=3847 GN=100795745 PE=4 SV=1	2	1	2	1	3	1
I1LJE2	Coatomer subunit gamma OS=Glycine max OX=3847 GN=100791801 PE=3 SV=1	2	3	2	3	3	3
C6SXW4	Proteasome subunit beta OS=Glycine max OX=3847 GN=100306084 PE=2 SV=1	2	2	2	2	2	2
I1N541	Uncharacterized protein OS=Glycine max OX=3847 GN=100783822 PE=3 SV=2	2	2	1	1	4	2
I1KEY6	26S proteasome non-ATPase regulatory subunit 2 homolog	2	2	2	2	2	2
A0A0R0IU60	Uncharacterized protein OS=Glycine max OX=3847 GN=100777674 PE=3 SV=1	2	2	2	2	3	2
C6T8E0	Uncharacterized protein OS=Glycine max OX=3847 GN=100778672 PE=2 SV=1	2	1	2	1	5	2
A0A0R4J3L5	Proteasome subunit alpha type OS=Glycine max OX=3847 GN=732640 PE=3 SV=1	2	1	2	1	2	1
I1JEL6	Uncharacterized protein OS=Glycine max OX=3847 GN=GLYMA_02G124500 PE=3 SV=1	2	1	1	1	4	1
A0A0R0FB78	Uncharacterized protein OS=Glycine max OX=3847 GN=100779679 PE=4 SV=1	2	2	2	2	4	3
Q42807	Stearoyl-[acyl-carrier-protein] 9-desaturase, chloroplastic	2	3	2	3	3	3
A0A0R0JEI9	Uncharacterized protein OS=Glycine max OX=3847 GN=GLYMA_06G094400 PE=3 SV=1	2	N/A	1	N/A	6	N/A
I1K7Q8	Uncharacterized protein OS=Glycine max OX=3847 GN=100813308 PE=4 SV=1	2	2	2	2	9	4
I1JXC1	Elongation factor Tu OS=Glycine max OX=3847 GN=100805960 PE=3 SV=1	2	1	2	1	2	1
A0A0R0LFB4	Dihydrolipoamide acetyltransferase component of pyruvate dehydrogenase complex	2	2	2	2	5	4
I1N898	Tubulin alpha chain OS=Glycine max OX=3847 GN=100796371 PE=3 SV=1	2	N/A	1	N/A	4	N/A
I1K694	Uncharacterized protein OS=Glycine max OX=3847 GN=100812565 PE=3 SV=1	2	N/A	2	N/A	2	N/A
I1KZJ0	Uncharacterized protein OS=Glycine max OX=3847 GN=100807267 PE=3 SV=1	2	2	2	2	3	4
A0A0R0IUS1	Serine hydroxymethyltransferase OS=Glycine max OX=3847 GN=100305380 PE=3 SV=1	2	1	2	1	4	1
I1J6K4	Uncharacterized protein OS=Glycine max OX=3847 GN=100808526 PE=3 SV=2	2	1	2	1	8	6
I1J5Y9	UDP-glucose 6-dehydrogenase OS=Glycine max OX=3847 GN=GLYMA_01G057100	2	1	1	1	4	1

C6TM03	Malate dehydrogenase OS=Glycine max OX=3847 GN=100799793 PE=2 SV=1	2	2	1	2	4	4
I1KDM8	Malate dehydrogenase OS=Glycine max OX=3847 GN=GLYMA_06G231500 PE=3 SV=1	2	2	1	1	2	5
I1KL72	Uncharacterized protein OS=Glycine max OX=3847 GN=100796791 PE=3 SV=1	2	N/A	1	N/A	3	N/A
C6T827	Pyruvate dehydrogenase E1 component subunit beta OS=Glycine max OX=3847	2	2	2	2	3	2
I1M5L7	Uncharacterized protein OS=Glycine max OX=3847 GN=100813658 PE=3 SV=1	2	2	2	2	2	2
I1KHP5	Pyruvate dehydrogenase E1 component subunit alpha OS=Glycine max OX=3847	2	1	2	1	5	1
I1K146	Uncharacterized protein OS=Glycine max OX=3847 GN=100796742 PE=4 SV=1	2	N/A	2	N/A	3	N/A
I1LB65	Importin subunit alpha OS=Glycine max OX=3847 GN=100789135 PE=3 SV=1	2	2	2	2	2	2
I1NH45	Peroxidase OS=Glycine max OX=3847 GN=100775837 PE=3 SV=1	2	2	2	2	9	10
A0A0R4J2N4	Pyruvate kinase OS=Glycine max OX=3847 GN=100776984 PE=3 SV=1	2	1	2	1	3	1
I1LNM2	NADH dehydrogenase subunit 9 OS=Glycine max OX=3847 GN=nad9 PE=3 SV=1	2	2	2	2	4	4
A5JVZ7	Superoxide dismutase OS=Glycine max OX=3847 GN=100785490 PE=2 SV=1	2	1	2	1	2	1
I1LUJ6	Uncharacterized protein OS=Glycine max OX=3847 GN=100793227 PE=4 SV=1	2	1	2	1	2	1
K7K7B4	Uncharacterized protein OS=Glycine max OX=3847 GN=GLYMA_02G092800 PE=3 SV=1	2	N/A	1	N/A	4	N/A
K7N4X3	Alpha-galactosidase OS=Glycine max OX=3847 GN=100797088 PE=3 SV=1	2	2	1	1	4	5
I1M2K9	Uncharacterized protein OS=Glycine max OX=3847 GN=GLYMA_13G254900 PE=3 SV=1	2	2	1	2	2	2
I1K135	Glyceraldehyde-3-phosphate dehydrogenase OS=Glycine max OX=3847 GN=100794095	2	N/A	1	N/A	7	N/A
I1KIV5	Uncharacterized protein OS=Glycine max OX=3847 GN=100799440 PE=4 SV=1	2	2	2	2	2	3
A0A0R0II89	Uncharacterized protein OS=Glycine max OX=3847 GN=GLYMA_08G068800 PE=3 SV=1	2	N/A	1	N/A	2	N/A
K7KGC7	Sucrose synthase OS=Glycine max OX=3847 GN=100793371 PE=3 SV=1	2	1	1	1	3	1
P50346	60S acidic ribosomal protein P0 OS=Glycine max OX=3847 PE=2 SV=1	2	1	2	1	2	1
I1K467	Uncharacterized protein OS=Glycine max OX=3847 GN=100805255 PE=3 SV=1	2	N/A	2	N/A	2	N/A
I1KBV2	Ribonuclease OS=Glycine max OX=3847 GN=100794481 PE=4 SV=1	2	N/A	2	N/A	2	N/A
C6SWS6	Uncharacterized protein OS=Glycine max OX=3847 GN=100305824 PE=2 SV=1	2	N/A	2	N/A	2	N/A
I1M5G8	Malic enzyme OS=Glycine max OX=3847 GN=100800844 PE=3 SV=1	2	3	2	2	6	5
I1N0I5	Uncharacterized protein OS=Glycine max OX=3847 GN=100786112 PE=3 SV=2	2	N/A	2	N/A	2	N/A
I1LMA5	Uncharacterized protein OS=Glycine max OX=3847 GN=100802040 PE=3 SV=1	2	N/A	2	N/A	2	N/A
I1MZ56	Endoglucanase OS=Glycine max OX=3847 GN=732658 PE=3 SV=1	2	N/A	1	N/A	6	N/A
I1JBV5	Pyruvate dehydrogenase E1 component subunit alpha OS=Glycine max OX=3847	2	N/A	2	N/A	2	N/A
O81413	Ferric leghemoglobin reductase-2 OS=Glycine max OX=3847 GN=547523 PE=2 SV=1	2	N/A	2	N/A	2	N/A
K7MAP3	Uncharacterized protein OS=Glycine max OX=3847 GN=GLYMA_15G102600 PE=3 SV=1	2	1	2	1	3	1
A0A0R0FQN0	Aspartate aminotransferase OS=Glycine max OX=3847 GN=547792 PE=4 SV=1	2	N/A	1	N/A	2	N/A
I1JBJ9	Endoglucanase OS=Glycine max OX=3847 GN=GLYMA_02G016400 PE=3 SV=1	2	2	1	2	8	8
A0A0R4J4A9	Uncharacterized protein OS=Glycine max OX=3847 GN=100795325 PE=4 SV=1	2	N/A	1	N/A	3	N/A
I1MGG7	Uncharacterized protein OS=Glycine max OX=3847 GN=100803176 PE=4 SV=1	2	N/A	2	N/A	2	N/A
C6TFG0	Uncharacterized protein OS=Glycine max OX=3847 GN=100810569 PE=2 SV=1	2	2	2	2	2	4

I1J732	Uncharacterized protein OS=Glycine max OX=3847 GN=GLYMA_01G106800 PE=3 SV=1	2	2	2	2	4	4
K7LK37	Pyruvate kinase OS=Glycine max OX=3847 GN=GLYMA_10G178900 PE=3 SV=1	2	1	2	1	2	1
C6T4Z6	Uncharacterized protein OS=Glycine max OX=3847 GN=100527647 PE=2 SV=1	2	1	2	1	2	1
I1MS58	Beta-galactosidase OS=Glycine max OX=3847 GN=GLYMA_17G047400 PE=3 SV=2	2	1	2	1	7	2
C6TGU0	DHAR class glutathione S-transferase OS=Glycine max OX=3847 GN=DHAR3 PE=2 SV=1	2	2	2	1	3	3
I1KQU5	Malate dehydrogenase OS=Glycine max OX=3847 GN=GLYMA_08G063800 PE=3 SV=1	2	N/A	1	N/A	3	N/A
A0A0R0H7D5	Uncharacterized protein OS=Glycine max OX=3847 GN=GLYMA_12G192400 PE=3 SV=1	2	N/A	2	N/A	2	N/A
C6THA9	Uncharacterized protein OS=Glycine max OX=3847 GN=100790835 PE=2 SV=1	2	2	2	2	4	4
I1MM57	40S ribosomal protein S6 OS=Glycine max OX=3847 GN=100792949 PE=3 SV=2	2	N/A	2	N/A	2	N/A
I1J8H1	Aconitate hydratase OS=Glycine max OX=3847 GN=100499625 PE=3 SV=1	2	4	1	1	3	5
I1JTB8	Glucose-6-phosphate isomerase OS=Glycine max OX=3847 GN=GLYMA_04G032600	2	N/A	2	N/A	2	N/A
C6SYA5	60S ribosomal protein L18a OS=Glycine max OX=3847 GN=100306174 PE=2 SV=1	2	N/A	2	N/A	3	N/A
P62302	40S ribosomal protein S13 OS=Glycine max OX=3847 GN=RPS13 PE=2 SV=1	2	1	2	1	2	2
I1JJB5	Uncharacterized protein OS=Glycine max OX=3847 GN=100799149 PE=4 SV=1	2	3	2	3	2	3
I1L171	Uncharacterized protein OS=Glycine max OX=3847 GN=100800020 PE=4 SV=1	2	1	1	1	2	1
C6TLT3	40S ribosomal protein S3a OS=Glycine max OX=3847 GN=100808747 PE=2 SV=1	2	N/A	2	N/A	4	N/A
A0A0R0I7V5	Uncharacterized protein OS=Glycine max OX=3847 GN=GLYMA_10G224200 PE=3 SV=1	2	N/A	2	N/A	2	N/A
C6T034	Uncharacterized protein OS=Glycine max OX=3847 GN=100500082 PE=2 SV=1	2	N/A	2	N/A	3	N/A
A0A0R0G6T3	Uncharacterized protein OS=Glycine max OX=3847 GN=GLYMA_15G038100 PE=3 SV=1	2	2	2	1	3	2
A0A0R0F5Q2	Uncharacterized protein OS=Glycine max OX=3847 GN=100809809 PE=4 SV=1	2	2	2	2	2	3
I1LDQ6	Uncharacterized protein OS=Glycine max OX=3847 GN=100787184 PE=4 SV=2	2	2	1	2	3	2
I1JWZ3	Plasma membrane ATPase OS=Glycine max OX=3847 GN=100818043 PE=3 SV=1	2	N/A	1	N/A	2	N/A
I1JIP5	T-complex protein 1 subunit eta OS=Glycine max OX=3847 GN=100784862 PE=3 SV=1	2	2	2	2	3	2
I1MM62	Beta-galactosidase OS=Glycine max OX=3847 GN=100795578 PE=3 SV=2	2	N/A	2	N/A	2	N/A
I1KJ35	Uncharacterized protein OS=Glycine max OX=3847 GN=100814740 PE=3 SV=1	2	2	2	2	3	2
A0A0R0FM59	Glucose-6-phosphate 1-dehydrogenase OS=Glycine max OX=3847 GN=100793462 PE=3	2	2	2	2	2	2
I1KW20	Uncharacterized protein OS=Glycine max OX=3847 GN=100791896 PE=4 SV=1	2	2	2	2	2	2
O80412	Mitochondrial phosphate transporter OS=Glycine max OX=3847 GN=548006 PE=2 SV=1	2	N/A	2	N/A	3	N/A
I1NFX7	D-3-phosphoglycerate dehydrogenase OS=Glycine max OX=3847 GN=100808771 PE=3 SV=1	2	2	2	2	3	3
I1LVJ0	S-adenosylmethionine synthase OS=Glycine max OX=3847 GN=100820392 PE=3 SV=1	2	N/A	2	N/A	2	N/A
I1JXP9	Uncharacterized protein OS=Glycine max OX=3847 GN=100796195 PE=3 SV=1	2	1	2	1	3	1
A0A0R0HHB6	Uncharacterized protein OS=Glycine max OX=3847 GN=GLYMA_11G146300 PE=3 SV=1	2	2	2	2	4	2
I1LWI3	Uncharacterized protein OS=Glycine max OX=3847 GN=100306559 PE=4 SV=1	2	1	2	1	2	1
A0A0R4J2L9	Malate dehydrogenase OS=Glycine max OX=3847 GN=GLYMA_02G005500 PE=3 SV=1	3	3	3	2	8	3
A0A0R0GHB3	Uncharacterized protein OS=Glycine max OX=3847 GN=100806710 PE=3 SV=1	3	2	1	1	8	5
A0A0R0F2Z4	Uncharacterized protein OS=Glycine max OX=3847 GN=100820155 PE=4 SV=1	3	3	3	3	8	4

I1MDV8	Uncharacterized protein OS=Glycine max OX=3847 GN=100817945 PE=3 SV=1	3	4	3	4	7	7
A0A0R0EHR6	6-phosphogluconate dehydrogenase, decarboxylating OS=Glycine max OX=3847	3	4	2	2	4	5
Q2I0H4	Glyceraldehyde-3-phosphate dehydrogenase OS=Glycine max OX=3847 GN=GAPC1	3	3	1	2	79	33
I1K2I1	Tubulin alpha chain OS=Glycine max OX=3847 GN=100787058 PE=3 SV=1	3	3	2	3	6	5
I1N8I7	Uncharacterized protein OS=Glycine max OX=3847 GN=GLYMA_19G121900 PE=4 SV=1	3	3	3	3	7	7
I1K5Y5	Malic enzyme OS=Glycine max OX=3847 GN=100782096 PE=3 SV=1	3	4	3	3	8	10
I1KVU0	Uncharacterized protein OS=Glycine max OX=3847 GN=GLYMA_08G224400 PE=3 SV=1	3	N/A	3	N/A	3	N/A
I1LWV7	Uncharacterized protein OS=Glycine max OX=3847 GN=100778357 PE=4 SV=1	3	N/A	2	N/A	3	N/A
I1NDI4	Uncharacterized protein OS=Glycine max OX=3847 GN=100815948 PE=3 SV=2	3	3	2	2	8	6
I1K3S4	Adenosylhomocysteinase OS=Glycine max OX=3847 GN=GLYMA_05G152000 PE=3 SV=1	3	2	3	2	5	3
I1N6A6	UDP-glucose 6-dehydrogenase OS=Glycine max OX=3847 GN=100810907 PE=3 SV=1	3	2	2	2	5	3
I1KD37	Uncharacterized protein OS=Glycine max OX=3847 GN=100816315 PE=4 SV=1	3	4	2	4	6	6
I1JDR2	Uncharacterized protein OS=Glycine max OX=3847 GN=100786978 PE=4 SV=1	3	2	3	2	4	3
I1KZK2	Uncharacterized protein OS=Glycine max OX=3847 GN=100809943 PE=3 SV=1	3	1	3	1	4	1
I1JED0	UDP-glucose 6-dehydrogenase OS=Glycine max OX=3847 GN=100799142 PE=3 SV=1	3	N/A	2	N/A	5	N/A
K7MJ32	Alpha-galactosidase OS=Glycine max OX=3847 GN=GLYMA_16G220400 PE=3 SV=1	3	3	2	2	5	8
I1JMB4	Glyceraldehyde-3-phosphate dehydrogenase OS=Glycine max OX=3847	3	1	3	1	6	2
A0A0R0IX14	Uncharacterized protein OS=Glycine max OX=3847 GN=100785346 PE=3 SV=1	3	N/A	2	N/A	3	N/A
I1JZP0	Uncharacterized protein OS=Glycine max OX=3847 GN=100783173 PE=4 SV=1	3	N/A	2	N/A	3	N/A
I1LUH6	Uncharacterized protein OS=Glycine max OX=3847 GN=100785990 PE=3 SV=1	3	2	1	1	3	2
I1JJM3	Fructose-bisphosphate aldolase OS=Glycine max OX=3847	3	3	1	1	7	7
A0A0R0HYT3	Uncharacterized protein OS=Glycine max OX=3847 GN=GLYMA_10G069600 PE=4 SV=1	3	2	3	1	5	4
C6TD56	Glyceraldehyde-3-phosphate dehydrogenase OS=Glycine max OX=3847 GN=100807342	3	2	1	1	10	9
C6T1V2	Uncharacterized protein OS=Glycine max OX=3847 GN=100500475 PE=2 SV=1	3	3	2	2	5	4
A0A0R0JNS2	Uncharacterized protein OS=Glycine max OX=3847 GN=GLYMA_06G317400 PE=3 SV=1	3	3	3	3	3	3
I1JCQ7	Succinate dehydrogenase [ubiquinone] flavoprotein subunit, mitochondrial	3	4	1	4	3	4
K7M3V5	Coatomer subunit beta' OS=Glycine max OX=3847 GN=100815780 PE=3 SV=1	3	1	3	1	3	1
I1K672	Uncharacterized protein OS=Glycine max OX=3847 GN=100805445 PE=4 SV=1	3	3	3	3	4	3
O48548	Aspartate aminotransferase OS=Glycine max OX=3847 GN=AAT PE=2 SV=1	3	2	2	2	5	4
I1MJN2	Uncharacterized protein OS=Glycine max OX=3847 GN=100789942 PE=3 SV=1	3	2	1	1	3	2
I1M984	Uncharacterized protein OS=Glycine max OX=3847 GN=100788164 PE=3 SV=1	3	2	3	2	4	4
A0A0R0GJT8	Uncharacterized protein OS=Glycine max OX=3847 GN=GLYMA_13G078600 PE=4 SV=1	3	3	1	1	6	8
I1LHR7	Succinate dehydrogenase [ubiquinone] flavoprotein subunit, mitochondrial OS=Glycine max OX=3847 GN=100818379 PE=3 SV=1	3	N/A	1	N/A	3	N/A
I1KVJ0	Plasma membrane ATPase OS=Glycine max OX=3847 GN=100796279 PE=3 SV=2	3	N/A	1	N/A	3	N/A
I1MPM3	Uncharacterized protein OS=Glycine max OX=3847 GN=100798235 PE=4 SV=2	3	1	2	1	3	1
I1MSP4	Plasma membrane ATPase OS=Glycine max OX=3847 GN=100816905 PE=3 SV=2	3	2	1	2	3	2

A0A0R4J4X6	Uncharacterized protein OS=Glycine max OX=3847 GN=GLYMA_13G176300 PE=3 SV=1	3	5	1	4	3	10
I1JDT9	Glucose-6-phosphate 1-dehydrogenase OS=Glycine max OX=3847	3	N/A	3	N/A	5	N/A
A0A0R0IR73	40S ribosomal protein SA OS=Glycine max OX=3847 GN=100786575 PE=3 SV=1	3	N/A	1	N/A	5	N/A
K7KYB1	Aconitate hydratase OS=Glycine max OX=3847 GN=100793264 PE=3 SV=1	3	4	1	1	6	5
A0A0R0FY49	Uncharacterized protein OS=Glycine max OX=3847 GN=GLYMA_15G088000 PE=3 SV=1	4	4	1	1	16	11
K7L2A0	Uncharacterized protein OS=Glycine max OX=3847 GN=102667572 PE=3 SV=1	4	N/A	2	N/A	9	N/A
I1LL24	Uncharacterized protein OS=Glycine max OX=3847 GN=100806471 PE=3 SV=1	4	2	3	2	8	3
A0A0R0GRY6	Uncharacterized protein OS=Glycine max OX=3847 GN=GLYMA_14G176900 PE=3 SV=1	4	4	2	2	11	10
I1L655	Uncharacterized protein OS=Glycine max OX=3847 GN=100814088 PE=3 SV=1	4	5	4	5	9	13
C6SZI8	Uncharacterized protein OS=Glycine max OX=3847 GN=100500031 PE=2 SV=1	4	4	1	1	6	6
I1MC13	Uncharacterized protein OS=Glycine max OX=3847 GN=100819526 PE=4 SV=1	4	3	4	3	4	3
I1KH24	Phospholipase D OS=Glycine max OX=3847 GN=100794849 PE=3 SV=1	4	5	4	5	5	6
A0A0R0FJR0	40S ribosomal protein SA OS=Glycine max OX=3847 GN=100805318 PE=3 SV=1	4	3	2	3	8	6
A0A0R0FDT6	Uncharacterized protein OS=Glycine max OX=3847 GN=100784882 PE=4 SV=1	4	1	2	1	5	1
I1JY29	Uncharacterized protein OS=Glycine max OX=3847 GN=100783318 PE=3 SV=1	4	3	4	3	9	7
Q06197	Isocitrate dehydrogenase [NADP] OS=Glycine max OX=3847 GN=IDH1 PE=1 SV=2	4	3	4	2	6	4
A0A0R4J5H1	Succinate-semialdehyde dehydrogenase OS=Glycine max OX=3847 GN=100808318	4	N/A	4	N/A	7	N/A
C6TGU2	Proteasome subunit alpha type OS=Glycine max OX=3847 GN=100796147 PE=2 SV=1	4	4	4	4	6	7
A0A0R4J5Y4	Uncharacterized protein OS=Glycine max OX=3847 GN=GLYMA_18G251300 PE=4 SV=1	4	N/A	1	N/A	6	N/A
I1JB52	Uncharacterized protein OS=Glycine max OX=3847 GN=100788766 PE=3 SV=1	4	N/A	4	N/A	4	N/A
I1M4T8	Uncharacterized protein OS=Glycine max OX=3847 GN=100780673 PE=4 SV=1	4	N/A	2	N/A	6	N/A
K7MJY8	Sucrose synthase OS=Glycine max OX=3847 GN=GLYMA_17G045800 PE=3 SV=1	4	1	3	1	14	6
I1JPX8	Fructose-bisphosphate aldolase OS=Glycine max OX=3847 GN=100527532 PE=3 SV=1	4	4	2	2	8	7
C6T4R9	Uncharacterized protein OS=Glycine max OX=3847 GN=100527579 PE=2 SV=1	4	N/A	4	N/A	10	N/A
I1LCI1	Uncharacterized protein OS=Glycine max OX=3847 GN=100818544 PE=3 SV=1	4	2	3	2	4	2
I1KZJ9	Uncharacterized protein OS=Glycine max OX=3847 GN=100810476 PE=4 SV=1	4	2	4	2	5	3
C6SZX7	Glutathione peroxidase OS=Glycine max OX=3847 GN=100306570 PE=2 SV=1	4	2	4	2	7	4
I1LKU1	Uncharacterized protein OS=Glycine max OX=3847 GN=100794780 PE=3 SV=1	4	1	4	1	4	1
A0A0R0L9T9	40S ribosomal protein S4 OS=Glycine max OX=3847 GN=100801792 PE=3 SV=1	4	5	4	5	4	6
A0A0R0F2E8	Uncharacterized protein OS=Glycine max OX=3847 GN=GLYMA_19G228800 PE=3 SV=1	5	4	2	1	7	6
A0A0R4J5M7	Uncharacterized protein OS=Glycine max OX=3847 GN=100818694 PE=4 SV=1	5	N/A	2	N/A	7	N/A
I1KPH6	6-phosphogluconate dehydrogenase, decarboxylating OS=Glycine max	5	3	4	1	5	4
A0A0R0KXC5	Uncharacterized protein OS=Glycine max OX=3847 GN=GLYMA_02G009600 PE=3 SV=1	5	N/A	5	N/A	9	N/A
I1LU53	Aconitate hydratase OS=Glycine max OX=3847 GN=100794803 PE=3 SV=1	5	7	3	1	7	11
I1JWK3	Uncharacterized protein OS=Glycine max OX=3847 GN=100776541 PE=3 SV=1	5	5	1	2	9	6
A0A0R0KPW1	Uncharacterized protein OS=Glycine max OX=3847 GN=GLYMA_03G114400 PE=4 SV=2	5	5	3	3	6	6

I1MJU7	Uncharacterized protein OS=Glycine max OX=3847 GN=100800698 PE=3 SV=1	5	5	5	5	5	6
I1NAI7	Uncharacterized protein OS=Glycine max OX=3847 GN=GLYMA_19G190900 PE=3 SV=1	6	6	3	3	39	29
A0A0R0K0U9	Uncharacterized protein OS=Glycine max OX=3847 GN=GLYMA_05G022200 PE=3 SV=1	6	2	5	2	6	3
A0A0R4J5A7	Uncharacterized protein OS=Glycine max OX=3847 GN=100786735 PE=3 SV=1	6	N/A	5	N/A	10	N/A
A0A0R0EX12	Histone H4 OS=Glycine max OX=3847 GN=100779904 PE=3 SV=1	6	6	6	6	12	12
I1LMS9	Clathrin heavy chain OS=Glycine max OX=3847 GN=100802224 PE=3 SV=1	6	7	6	7	8	10
K7LQC6	Uncharacterized protein OS=Glycine max OX=3847 GN=100805946 PE=4 SV=1	6	N/A	6	N/A	6	N/A
A0A0R4J5Z8	Uncharacterized protein OS=Glycine max OX=3847 GN=GLYMA_19G114500 PE=3 SV=1	6	5	2	2	9	5
I1MT10	Uncharacterized protein OS=Glycine max OX=3847 GN=100782173 PE=3 SV=1	7	8	1	1	25	23
I1KZK0	Uncharacterized protein OS=Glycine max OX=3847 GN=GLYMA_08G363800 PE=3 SV=1	7	5	5	3	17	14
I1KU21	Uncharacterized protein OS=Glycine max OX=3847 GN=100804251 PE=4 SV=1	7	5	7	5	10	12
A0A0R4J4C8	ATP synthase subunit beta OS=Glycine max OX=3847 GN=100797948 PE=3 SV=1	8	10	6	8	23	23
A0A0R0FM97	Elongation factor 1-alpha OS=Glycine max OX=3847 GN=100801611 PE=3 SV=1	8	N/A	1	N/A	31	N/A
A0A0R4J4C3	Elongation factor 1-alpha OS=Glycine max OX=3847 GN=100776330 PE=3 SV=1	8	9	1	3	17	20
I1JT28	Tubulin beta chain OS=Glycine max OX=3847 GN=100781525 PE=3 SV=1	8	8	2	2	15	11
O23960	Biotin carboxylase OS=Glycine max OX=3847 GN=accC-2 PE=2 SV=1	8	5	8	5	13	9
Q01915	ATP synthase subunit alpha, mitochondrial OS=Glycine max OX=3847 GN=ATPA PE=3 SV=1	9	7	9	6	27	17
I1L0G9	Tubulin beta chain OS=Glycine max OX=3847 GN=100818878 PE=3 SV=1	9	10	3	4	20	15
I1MQ89	Uncharacterized protein OS=Glycine max OX=3847 GN=100787663 PE=3 SV=1	10	8	7	5	51	38
I1MC31	Uncharacterized protein OS=Glycine max OX=3847 GN=100779976 PE=3 SV=1	10	9	9	8	19	18
D6C500	HSP90-2 OS=Glycine max OX=3847 GN=100819568 PE=2 SV=1	10	8	3	1	26	19
I1M3L8	Uncharacterized protein OS=Glycine max OX=3847 GN=GLYMA_13G290700 PE=3 SV=2	10	10	2	1	16	14
I1KEN4	Uncharacterized protein OS=Glycine max OX=3847 GN=100807407 PE=3 SV=1	10	10	2	1	16	14
K7LZH1	Uncharacterized protein OS=Glycine max OX=3847 GN=GLYMA_13G130900 PE=3 SV=1	11	6	1	1	31	16
A0A0R0FH00	Uncharacterized protein OS=Glycine max OX=3847 GN=100787543 PE=3 SV=1	11	N/A	0	N/A	30	N/A
I1JG11	Uncharacterized protein OS=Glycine max OX=3847 GN=100813210 PE=3 SV=1	11	N/A	2	N/A	27	N/A
I1K4W1	Uncharacterized protein OS=Glycine max OX=3847 GN=GLYMA_05G188800 PE=3 SV=1	12	10	3	2	25	32
K7MVD3	Uncharacterized protein OS=Glycine max OX=3847 GN=100789485 PE=3 SV=1	13	8	0	0	38	24
A0A0R4J4D6	Uncharacterized protein OS=Glycine max OX=3847 GN=100812707 PE=3 SV=1	13	11	1	3	35	29
I1MPI1	Uncharacterized protein OS=Glycine max OX=3847 GN=100784477 PE=3 SV=1	13	13	1	1	31	22
I1KPN3	Uncharacterized protein OS=Glycine max OX=3847 GN=547838 PE=3 SV=1	15	N/A	13	N/A	41	N/A
I1L314	Heat shock protein 90-1 OS=Glycine max OX=3847 GN=100811234 PE=2 SV=1	15	15	2	2	36	27
I1K670	Uncharacterized protein OS=Glycine max OX=3847 GN=GLYMA_05G219400 PE=3 SV=1	N/A	13	N/A	11	N/A	28
C6T7Y1	Uncharacterized protein OS=Glycine max OX=3847 GN=100807341 PE=2 SV=1	N/A	9	N/A	1	N/A	31
A0A0R4J626	Uncharacterized protein OS=Glycine max OX=3847 GN=100777767 PE=3 SV=1	N/A	8	N/A	0	N/A	22
A0A0R0JTV5	Elongation factor 1-alpha OS=Glycine max OX=3847 GN=GLYMA_05G114900 PE=3 SV=1	N/A	7	N/A	1	N/A	20

I1KDL4	Phosphoenolpyruvate carboxylase OS=Glycine max OX=3847 GN=Gmppc2 PE=2 SV=1	N/A	8	N/A	1	N/A	10
A0A0R4J4Z2	Uncharacterized protein OS=Glycine max OX=3847 GN=GLYMA_13G106200 PE=3 SV=1	N/A	7	N/A	7	N/A	9
I1LRQ4	Aconitate hydratase OS=Glycine max OX=3847 GN=100778506 PE=3 SV=1	N/A	6	N/A	1	N/A	9
C6SXM4	Uncharacterized protein OS=Glycine max OX=3847 GN=100807631 PE=2 SV=1	N/A	6	N/A	6	N/A	7
K7M629	Uncharacterized protein OS=Glycine max OX=3847 GN=GLYMA_14G105300 PE=4 SV=1	N/A	2	N/A	1	N/A	6
C6SXX2	Uncharacterized protein OS=Glycine max OX=3847 GN=100794152 PE=2 SV=1	N/A	4	N/A	4	N/A	7
A0A0R0IRH4	Phosphoglycerate kinase OS=Glycine max OX=3847 GN=GLYMA_08G165400 PE=3 SV=1	N/A	3	N/A	3	N/A	4
A0A0R0F5U2	Uncharacterized protein OS=Glycine max OX=3847 GN=100782753 PE=4 SV=1	N/A	3	N/A	3	N/A	4
C6TGD9	Uncharacterized protein OS=Glycine max OX=3847 GN=100814078 PE=2 SV=1	N/A	2	N/A	1	N/A	5
I1MJH1	Isocitrate dehydrogenase [NADP] OS=Glycine max OX=3847 GN=100816688 PE=3 SV=1	N/A	3	N/A	2	N/A	4
I1KG34	Uncharacterized protein OS=Glycine max OX=3847 GN=GLYMA_07G002300 PE=3 SV=1	N/A	2	N/A	2	N/A	5
I1KTW3	Succinate-semialdehyde dehydrogenase OS=Glycine max OX=3847	N/A	3	N/A	3	N/A	6
C6TAB7	Uncharacterized protein OS=Glycine max OX=3847 GN=100803287 PE=2 SV=1	N/A	2	N/A	2	N/A	4
A0A0R0KF64	Uncharacterized protein OS=Glycine max OX=3847 GN=100125585 PE=3 SV=1	N/A	1	N/A	1	N/A	2
I1JXA0	Uncharacterized protein OS=Glycine max OX=3847 GN=100798680 PE=4 SV=1	N/A	4	N/A	4	N/A	5
Q2PMQ9	Photosystem II CP47 reaction center protein OS=Glycine max OX=3847 GN=psbB PE=3 SV=1	N/A	3	N/A	3	N/A	4
I1L068	Uncharacterized protein OS=Glycine max OX=3847 GN=100784816 PE=4 SV=1	N/A	3	N/A	3	N/A	3
I1LDE9	Uncharacterized protein OS=Glycine max OX=3847 GN=100806256 PE=3 SV=1	N/A	3	N/A	3	N/A	3
K7LWI4	ATP synthase subunit alpha OS=Glycine max OX=3847	N/A	2	N/A	1	N/A	2
Q2PMU2	Photosystem I P700 chlorophyll a apoprotein A2 OS=Glycine max OX=3847	N/A	2	N/A	2	N/A	2
A0A0R4J3Y6	Uncharacterized protein OS=Glycine max OX=3847 GN=GLYMA_08G224500 PE=3 SV=1	N/A	2	N/A	1	N/A	2
I1LEQ8	Fructose-bisphosphate aldolase OS=Glycine max OX=3847 GN=100802732 PE=3 SV=1	N/A	2	N/A	2	N/A	2
I1NJ59	DHAR class glutathione S-transferase OS=Glycine max OX=3847 GN=DHAR4 PE=2 SV=1	N/A	2	N/A	1	N/A	3
C6SVT8	Uncharacterized protein OS=Glycine max OX=3847 GN=100305599 PE=2 SV=1	N/A	2	N/A	1	N/A	4
I1JGP8	Uncharacterized protein OS=Glycine max OX=3847 GN=100787686 PE=3 SV=1	N/A	2	N/A	2	N/A	2
I1K1I6	Isocitrate dehydrogenase [NADP] OS=Glycine max OX=3847 GN=100819151 PE=3 SV=2	N/A	2	N/A	1	N/A	2
I1LZN1	Transmembrane 9 superfamily member OS=Glycine max OX=3847	N/A	1	N/A	1	N/A	1
I1KD09	ATP synthase subunit gamma OS=Glycine max OX=3847 GN=100813657 PE=3 SV=1	N/A	1	N/A	1	N/A	1
A0A0R0HUK1	Uncharacterized protein OS=Glycine max OX=3847 GN=100818032 PE=3 SV=1	N/A	2	N/A	2	N/A	3
I1MEK0	Eukaryotic translation initiation factor 3 subunit D OS=Glycine max OX=3847	N/A	1	N/A	1	N/A	1
A0A0R0EPH6	Uncharacterized protein OS=Glycine max OX=3847 GN=100819824 PE=3 SV=1	N/A	2	N/A	1	N/A	2
I1LA42	Uncharacterized protein OS=Glycine max OX=3847 GN=547729 PE=4 SV=1	N/A	2	N/A	2	N/A	2
A0A0R4J532	Uncharacterized protein OS=Glycine max OX=3847 GN=100305601 PE=4 SV=1	N/A	2	N/A	2	N/A	3
I1LSM4	Thiamine thiazole synthase, chloroplastic OS=Glycine max OX=3847 GN=THI1 PE=3 SV=1	N/A	1	N/A	1	N/A	1

Q2PMT9	Photosystem II CP43 reaction center protein OS=Glycine max OX=3847 GN=psbC PE=3 SV=1	N/A	2	N/A	2	N/A	2
B2BF98	40S ribosomal protein S6 OS=Glycine max OX=3847 GN=547746 PE=3 SV=1	N/A	2	N/A	2	N/A	2
I1KNG0	Uncharacterized protein OS=Glycine max OX=3847 GN=100779619 PE=3 SV=1	N/A	1	N/A	1	N/A	2
A0A0R4J5I3	Chlorophyll a-b binding protein, chloroplastic OS=Glycine max	N/A	2	N/A	2	N/A	4
A0A0R0EXW6	Uncharacterized protein OS=Glycine max OX=3847 GN=100776168 PE=3 SV=1	N/A	2	N/A	2	N/A	2
I1KEA1	Uncharacterized protein OS=Glycine max OX=3847 GN=100779962 PE=4 SV=1	N/A	1	N/A	1	N/A	1
K7L3L7	Importin subunit alpha OS=Glycine max OX=3847 GN=100813320 PE=3 SV=1	N/A	1	N/A	1	N/A	1
I7FST9	Protein disulfide-isomerase OS=Glycine max OX=3847 GN=Gm pdil-1 PE=2 SV=1	N/A	1	N/A	1	N/A	2
A0A0R0HLG7	Uncharacterized protein (Fragment) OS=Glycine max OX=3847	N/A	1	N/A	1	N/A	2
A0A0R0JI98	Uncharacterized protein OS=Glycine max OX=3847 GN=100777627 PE=3 SV=1	N/A	2	N/A	2	N/A	4
A0A0R0EC54	Uncharacterized protein OS=Glycine max OX=3847 GN=GLYMA_20G167600 PE=3 SV=1	N/A	2	N/A	2	N/A	3
I1N6A5	Alpha-1,4 glucan phosphorylase OS=Glycine max OX=3847 GN=100806646 PE=3 SV=1	N/A	1	N/A	1	N/A	1
I1KXF0	Proteasome subunit beta OS=Glycine max OX=3847 GN=100810291 PE=3 SV=1	N/A	1	N/A	1	N/A	1
I1K2Z9	Uncharacterized protein OS=Glycine max OX=3847 GN=100785835 PE=4 SV=1	N/A	1	N/A	1	N/A	1
I1L1U2	Sucrose synthase OS=Glycine max OX=3847 GN=100806761 PE=3 SV=1	N/A	1	N/A	1	N/A	2
I1JSS2	Dolichyl-diphosphooligosaccharide--protein glycosyltransferase subunit 1	N/A	1	N/A	1	N/A	2
I1LXZ7	Malate dehydrogenase OS=Glycine max OX=3847 GN=100783188 PE=3 SV=1	N/A	2	N/A	1	N/A	2
I1LQ57	Dolichyl-diphosphooligosaccharide--protein glycosyltransferase 48 kDa subunit	N/A	1	N/A	1	N/A	1
I1KGY6	Uncharacterized protein OS=Glycine max OX=3847 GN=100803691 PE=3 SV=1	N/A	1	N/A	1	N/A	1
I1L8L2	Uncharacterized protein OS=Glycine max OX=3847 GN=GLYMA_10G044000 PE=3 SV=1	N/A	1	N/A	1	N/A	1
I1MHR0	S-adenosylmethionine synthase OS=Glycine max OX=3847 GN=100787994 PE=3 SV=1	N/A	1	N/A	1	N/A	1
I1JUQ0	Uncharacterized protein OS=Glycine max OX=3847 GN=GLYMA_04G078200 PE=4 SV=1	N/A	2	N/A	2	N/A	2
I1JR22	Uncharacterized protein OS=Glycine max OX=3847 GN=100787551 PE=4 SV=1	N/A	1	N/A	1	N/A	1
A0A0R0GJV8	Uncharacterized protein OS=Glycine max OX=3847 GN=GLYMA_14G139600 PE=4 SV=1	N/A	1	N/A	1	N/A	1
A0A0R4J321	Proteasome subunit beta OS=Glycine max OX=3847 GN=100793551 PE=3 SV=1	N/A	1	N/A	1	N/A	1
I1L7A3	Peroxidase OS=Glycine max OX=3847 GN=100796311 PE=3 SV=1	N/A	1	N/A	1	N/A	2
I1MUM7	Malate synthase OS=Glycine max OX=3847 GN=GLYMA_17G128000 PE=3 SV=1	N/A	1	N/A	1	N/A	2
A0A0R4J336	Uncharacterized protein OS=Glycine max OX=3847 GN=GLYMA_04G045000 PE=3 SV=1	N/A	1	N/A	1	N/A	1
I1KRP1	Transmembrane 9 superfamily member OS=Glycine max OX=3847	N/A	1	N/A	1	N/A	1
I1JTZ0	Aminopeptidase OS=Glycine max OX=3847 GN=100811437 PE=3 SV=1	N/A	1	N/A	1	N/A	1
I1KG99	Uncharacterized protein OS=Glycine max OX=3847 GN=547466 PE=4 SV=1	N/A	1	N/A	1	N/A	1
C6SXB4	Uncharacterized protein OS=Glycine max OX=3847 GN=100499846 PE=2 SV=1	N/A	1	N/A	1	N/A	1
C6TI64	40S ribosomal protein S3a OS=Glycine max OX=3847 GN=100813273 PE=2 SV=1	N/A	1	N/A	1	N/A	1
A0A0R0H3H8	Uncharacterized protein OS=Glycine max OX=3847 GN=GLYMA_12G096400 PE=4 SV=1	N/A	1	N/A	1	N/A	1
Q41219	Leghemoglobin reductase OS=Glycine max OX=3847 GN=FLBR PE=1 SV=1	N/A	1	N/A	1	N/A	1
A0A0R0FI12	Uncharacterized protein OS=Glycine max OX=3847 GN=100801978 PE=4 SV=1	N/A	1	N/A	1	N/A	1

K7LLZ6	Uncharacterized protein OS=Glycine max OX=3847 GN=100805723 PE=4 SV=1	N/A	1	N/A	1	N/A	8
I1KUE5	Chlorophyll a-b binding protein, chloroplastic	N/A	1	N/A	1	N/A	1
Q2PMT8	Photosystem II D2 protein OS=Glycine max OX=3847 GN=psbD PE=3 SV=1	N/A	1	N/A	1	N/A	1
K7MTV5	Uncharacterized protein OS=Glycine max OX=3847 GN=100802703 PE=3 SV=1	N/A	1	N/A	1	N/A	1
I1KMS8	Uncharacterized protein OS=Glycine max OX=3847 GN=GLYMA_07G241600 PE=4 SV=1	N/A	1	N/A	1	N/A	1
C6SX87	Uncharacterized protein OS=Glycine max OX=3847 GN=100305937 PE=2 SV=1	N/A	1	N/A	1	N/A	1
C6T4P6	Uncharacterized protein OS=Glycine max OX=3847 GN=100527559 PE=2 SV=1	N/A	1	N/A	1	N/A	1
I1LZ51	Uncharacterized protein OS=Glycine max OX=3847 GN=100791677 PE=3 SV=1	N/A	1	N/A	1	N/A	1
A0A0R0FVN7	Uncharacterized protein OS=Glycine max OX=3847 GN=GLYMA_16G017200 PE=4 SV=1	N/A	1	N/A	1	N/A	1
I1LKZ2	Uncharacterized protein OS=Glycine max OX=3847 GN=100815376 PE=4 SV=1	N/A	1	N/A	1	N/A	1
C6SVC4	Uncharacterized protein OS=Glycine max OX=3847 GN=100305498 PE=2 SV=1	N/A	1	N/A	1	N/A	1
A0A0R0EKG7	Uncharacterized protein OS=Glycine max OX=3847 GN=GLYMA_20G124000 PE=4 SV=1	N/A	1	N/A	1	N/A	1
I1LHX5	Beta-galactosidase OS=Glycine max OX=3847 GN=100798501 PE=3 SV=1	N/A	1	N/A	1	N/A	1
A0A0R4J389	Uncharacterized protein OS=Glycine max OX=3847 GN=100807732 PE=4 SV=1	N/A	1	N/A	1	N/A	1
I1JCF7	Alpha-mannosidase OS=Glycine max OX=3847 GN=100775419 PE=3 SV=1	N/A	1	N/A	1	N/A	1
K7K9Q0	Uncharacterized protein OS=Glycine max OX=3847 GN=100777531 PE=4 SV=1	N/A	1	N/A	1	N/A	1
A0A0R0FMX4	Uncharacterized protein OS=Glycine max OX=3847 GN=GLYMA_16G082500 PE=4 SV=1	N/A	1	N/A	1	N/A	1
A0A0R0GVM 5	Xyloglucan endotransglucosylase/hydrolase	N/A	1	N/A	1	N/A	1
I1JXS8	Ribonuclease OS=Glycine max OX=3847 GN=100802766 PE=4 SV=1	N/A	1	N/A	1	N/A	1
I1JFV1	Serine/threonine-protein phosphatase OS=Glycine max OX=3847 GN=100801108 PE=3 SV=1	N/A	1	N/A	1	N/A	1
I1KTD4	Uncharacterized protein OS=Glycine max OX=3847 GN=100791893 PE=4 SV=2	N/A	1	N/A	1	N/A	1
A0A0R0K7Z5	Uncharacterized protein (Fragment) OS=Glycine max	N/A	1	N/A	1	N/A	1
I1LIH0	Uncharacterized protein OS=Glycine max OX=3847 GN=100789863 PE=4 SV=2	N/A	1	N/A	1	N/A	1
I1MBZ0	Uncharacterized protein OS=Glycine max OX=3847 GN=100811344 PE=4 SV=1	N/A	1	N/A	1	N/A	1
I1JBQ0	Uncharacterized protein OS=Glycine max OX=3847 GN=100789295 PE=4 SV=1	N/A	1	N/A	1	N/A	1
I1KQ42	Uncharacterized protein OS=Glycine max OX=3847 GN=100813160 PE=3 SV=1	N/A	1	N/A	1	N/A	1
A0A0R0IM08	Uncharacterized protein OS=Glycine max OX=3847 GN=GLYMA_08G015300 PE=3 SV=1	N/A	1	N/A	1	N/A	1
I1MTV4	Uncharacterized protein OS=Glycine max OX=3847 GN=100817791 PE=4 SV=1	N/A	1	N/A	1	N/A	1
C6SW24	Uncharacterized protein OS=Glycine max OX=3847 GN=100499738 PE=2 SV=1	N/A	1	N/A	1	N/A	1
I1KY83	Calcium-dependent protein kinase OS=Glycine max OX=3847 GN=547888 PE=4 SV=1	N/A	1	N/A	1	N/A	1
I1JQD4	Uncharacterized protein OS=Glycine max OX=3847 GN=GLYMA_03G205800 PE=3 SV=1	N/A	1	N/A	1	N/A	1
I1K4K1	Uncharacterized protein OS=Glycine max OX=3847 GN=100797102 PE=4 SV=1	N/A	1	N/A	1	N/A	1
I1KV60	RuvB-like helicase OS=Glycine max OX=3847 GN=100809596 PE=3 SV=1	N/A	1	N/A	1	N/A	1
K7KDG8	Uncharacterized protein OS=Glycine max OX=3847 GN=GLYMA_03G077700 PE=4 SV=1	N/A	1	N/A	1	N/A	1

A0A0R0HNY6	Uncharacterized protein OS=Glycine max OX=3847 GN=GLYMA_10G036400 PE=4 SV=1	N/A	1	N/A	1	N/A	1
K7K6W3	Uncharacterized protein OS=Glycine max OX=3847 GN=102662858 PE=4 SV=1	N/A	1	N/A	1	N/A	1
I1KPJ5	Nucleoside diphosphate kinase OS=Glycine max OX=3847	N/A	1	N/A	1	N/A	1
I1K6E7	Uncharacterized protein OS=Glycine max OX=3847 GN=GLYMA_05G211100 PE=4 SV=1	N/A	1	N/A	1	N/A	2
I1K8Q7	Uncharacterized protein OS=Glycine max OX=3847 GN=100792733 PE=4 SV=1	N/A	1	N/A	1	N/A	1
I1M5U8	Uncharacterized protein OS=Glycine max OX=3847 GN=100804026 PE=4 SV=1	N/A	1	N/A	1	N/A	1
A0A0R0G947	Uncharacterized protein OS=Glycine max OX=3847 GN=GLYMA_14G030000 PE=3 SV=1	N/A	1	N/A	1	N/A	1
I1KWH3	Uncharacterized protein OS=Glycine max OX=3847 GN=100781248 PE=4 SV=1	N/A	1	N/A	1	N/A	2
A0A0R0J154	Uncharacterized protein OS=Glycine max OX=3847 GN=100798414 PE=3 SV=1	N/A	1	N/A	1	N/A	1
I1K622	Uncharacterized protein OS=Glycine max OX=3847 GN=100790605 PE=3 SV=1	N/A	1	N/A	1	N/A	1
I1KAD3	Uncharacterized protein OS=Glycine max OX=3847 GN=100791865 PE=4 SV=2	N/A	1	N/A	1	N/A	1
I1KNM4	Uncharacterized protein OS=Glycine max OX=3847 GN=100806884 PE=4 SV=2	N/A	1	N/A	1	N/A	3
I1L056	Uncharacterized protein OS=Glycine max OX=3847 GN=100803933 PE=4 SV=2	N/A	1	N/A	1	N/A	3
I1JKP7	3-phosphoshikimate 1-carboxyvinyltransferase OS=Glycine max OX=3847	N/A	1	N/A	1	N/A	1
I1MUR2	Uncharacterized protein OS=Glycine max OX=3847 GN=100778423 PE=3 SV=1	N/A	1	N/A	1	N/A	1
I1KGH6	Uncharacterized protein OS=Glycine max OX=3847 GN=100810990 PE=3 SV=1	N/A	1	N/A	1	N/A	2
I1JKC3	Malic enzyme OS=Glycine max OX=3847 GN=100811081 PE=3 SV=2	N/A	1	N/A	1	N/A	1
I1JV02	Uncharacterized protein OS=Glycine max OX=3847 GN=100778495 PE=4 SV=1	N/A	1	N/A	1	N/A	1
I1LFG6	Uncharacterized protein OS=Glycine max OX=3847 GN=GLYMA_10G293800 PE=3 SV=1	N/A	1	N/A	1	N/A	1
I1L1N3	Uncharacterized protein OS=Glycine max OX=3847 GN=GLYMA_09G068000 PE=3 SV=1	N/A	1	N/A	1	N/A	1
I1LSY9	Uncharacterized protein OS=Glycine max OX=3847 GN=100805067 PE=4 SV=1	N/A	1	N/A	1	N/A	1
A0A0R0FS66	Uncharacterized protein OS=Glycine max OX=3847 GN=100810148 PE=4 SV=1	N/A	1	N/A	1	N/A	1
I1J4G6	Uncharacterized protein OS=Glycine max OX=3847 GN=100796303 PE=3 SV=1	N/A	1	N/A	1	N/A	1
I1K3D9	Uncharacterized protein OS=Glycine max OX=3847 GN=100776929 PE=4 SV=1	N/A	1	N/A	1	N/A	1
I1NI06	Uncharacterized protein OS=Glycine max OX=3847 GN=100786882 PE=4 SV=1	N/A	1	N/A	1	N/A	1
I1L1X0	Pectinesterase OS=Glycine max OX=3847 GN=100801813 PE=3 SV=1	N/A	1	N/A	1	N/A	1
I1JIU4	Uncharacterized protein OS=Glycine max OX=3847 GN=100800203 PE=4 SV=1	N/A	1	N/A	1	N/A	1
I1KB28	Uncharacterized protein OS=Glycine max OX=3847 GN=100807937 PE=4 SV=1	N/A	1	N/A	1	N/A	1
I1K5X6	Lactoylglutathione lyase OS=Glycine max OX=3847 GN=100780463 PE=3 SV=1	N/A	1	N/A	1	N/A	1
I1LNE6	T-complex protein 1 subunit epsilon OS=Glycine max OX=3847 GN=100817323 PE=3 SV=1	N/A	1	N/A	1	N/A	1
I1LRE2	DNA polymerase epsilon catalytic subunit OS=Glycine max OX=3847	N/A	1	N/A	1	N/A	1
K7MMG2	Uncharacterized protein OS=Glycine max OX=3847 GN=100810517 PE=4 SV=1	N/A	1	N/A	1	N/A	1
I1KXZ0	Phi class glutathione S-transferase OS=Glycine max OX=3847 GN=GSTF6 PE=2 SV=1	N/A	1	N/A	1	N/A	1
I1JPY7	Uncharacterized protein OS=Glycine max OX=3847 GN=100816963 PE=4 SV=1	N/A	1	N/A	1	N/A	1
Q1W376	Phosphomannomutase OS=Glycine max OX=3847 PE=2 SV=1	N/A	1	N/A	1	N/A	1
I1JT76	Beta-galactosidase OS=Glycine max OX=3847 GN=100795144 PE=3 SV=2	N/A	1	N/A	1	N/A	1

I1J5A4	Uncharacterized protein OS=Glycine max OX=3847 GN=100809428 PE=4 SV=1	N/A	1	N/A	1	N/A	1
C6SYH7	Small nuclear ribonucleoprotein E OS=Glycine max OX=3847 GN=100807929 PE=2 SV=1	N/A	1	N/A	1	N/A	1
I1K5B4	Uncharacterized protein OS=Glycine max OX=3847 GN=GLYMA_05G246700 PE=4 SV=2	N/A	1	N/A	1	N/A	1
I1N8V4	Uncharacterized protein OS=Glycine max OX=3847 GN=100816616 PE=4 SV=1	N/A	1	N/A	1	N/A	1

The protein ID was obtained through blasting against soybean protein database. “PSM” means peptide spectrum matching and was used to estimate the protein abundance. “N/A” means not assigned.
